# A layout framework for genome-wide multiple sequence alignment graphs

**DOI:** 10.3389/fbinf.2024.1358374

**Published:** 2024-08-16

**Authors:** Jeremias Schebera, Dirk Zeckzer, Daniel Wiegreffe

**Affiliations:** ^1^ Image and Signal Processing Group, Institute for Computer Science, Leipzig University, Leipzig, Germany; ^2^ Center for Scalable Data Analytics and Artificial Intelligence (ScaDS.AI) Dresden/Leipzig, Leipzig University, Leipzig, Germany

**Keywords:** genome analysis, multiple sequence alignment, graph drawing, visualization, genome comparison

## Abstract

Sequence alignments are often used to analyze genomic data. However, such alignments are often only calculated and compared on small sequence intervals for analysis purposes. When comparing longer sequences, these are usually divided into shorter sequence intervals for better alignment results. This usually means that the order context of the original sequence is lost. To prevent this, it is possible to use a graph structure to represent the order of the original sequence on the alignment blocks. The visualization of these graph structures can provide insights into the structural variations of genomes in a semi-global context. In this paper, we propose a new graph drawing framework for representing gMSA data. We produce a hierarchical graph layout that supports the comparative analysis of genomes. Based on a reference, the differences and similarities of the different genome orders are visualized. In this work, we present a complete graph drawing framework for gMSA graphs together with the respective algorithms for each of the steps. Additionally, we provide a prototype and an example data set for analyzing gMSA graphs. Based on this data set, we demonstrate the functionalities of the framework using two examples.

## 1 Introduction

The quality and the throughput of sequencing technologies, and thus the variety of data, have continuously increased in recent years (Genomes Project Consortium et al., 2015; Goodwin et al., 2016; Hickey et al., 2023). However, there is a large gap between sequence determination and sequence analysis (Gärtner et al., 2018). Especially, the capabilities for visually comparing sequences provided by the tools are insufficient, as most tools available today are designed for comparing closely related sequences or sequences of a limited length, only.

Multiple sequence alignments (MSAs) are a central procedure in the field of genetic information analysis (Albers et al., 2011). Sequence alignments are commonly visualized using dot plots, synteny views, and parallel coordinate views (Albers et al., 2011). Thereby, the focus is on visualizing local details in the alignments and not global trends. In the MSAs of higher animals and plants, the alignment block size is usually smaller than the size of individual genes (Gärtner et al., 2018). Consequently, it is difficult to analyze the MSAs from a more global perspective on rearrangements like the inversion, the translocation, or the duplication of a sequence with the traditionally used visualization approaches. On the other hand, there are approaches to compare whole genomes with genome-wide multiple sequence alignments (gMSAs). The visualizations showing global trends of such gMSAs are aggregated and are often restricted to only two sequences. As a result, rearrangements are barely visible in these global approaches.

Recently, research efforts and successes in pangenomes have increased (Consortium, 2016; Liao et al., 2023). In a pangenome similarities and differences are usually summarized in the form of a common reference genome assembly for a selection of individuals of one species. This addresses the problem that reference-based comparisons, such as classical MSAs, always have a bias towards the reference. Pangemones are usually represented by graph models (Consortium, 2016). The intervals of a pangenome can be visualized with a graph layout, e.g., by Sequence Tube Maps (Beyer et al., 2019), to compare the different sequences of the individuals used to generate the pangenome.

However, it would be also interesting to investigate variations between different species using a gMSA (Gärtner et al., 2018), especially if an artificially generated common coordinate system can be used as an additional order to counteract the bias toward the reference (Gärtner et al., 2018). Therefore, our overarching objective is to facilitate the examination of variations in the sequences of a gMSA from a semi-global perspective. For each original sequence in the MSA (e.g., a chromosome or contig), a total order of the alignment blocks can be found that represents the original sequence with the underlying sequence intervals. The sum of these alignment block sequences can be represented as a directed multi-graph (gMSA graph).

Here, we propose a graph layout framework that supports the visual comparative analysis of such gMSA graphs using the graph data basis produced by Gärtner et al. (Gärtner et al., 2018). This layout framework is based on the Sugiyama framework (Sugiyama et al., 1981) that was developed for directed acyclic graphs (DAGs). The Sugiyama framework consists of individual steps, for each of which several algorithms exist. However, most of the existing algorithms do not lead to layouts that meet the requirement, that the sequences forming a gMSA graph can be compared easily. Therefore, we propose new and tailored algorithms for most of the steps.

Our contribution is the description of the adapted Sugiyama framework to draw gMSA graphs to support the visual comparative analysis from a semi-global perspective and a prototype to demonstrate the results.

First, related work, important definitions, and fundamentals for both the biological and graph-based aspects are presented (Section 2 and 2.1). Then, the design criteria for the graph layout are presented (Section 2.2). Next, the entire framework from the input data to the final graph layout is described (Section 2.3). Two examples are provided (Section 3) followed by a discussion (Section 4).

## 2 Methods

Comparative genomics is the field of research in which two or more genomes are analyzed based on their *conservation* and *synteny* (Nusrat et al., 2019), The analysis of conservation consists of finding sequence intervals with a high degree of similarity in the genomes (e.g., with an alignment). Synteny refers to analyzing if the location, order, proximity, and orientation of the sequence intervals are similar in the compared sequences.

Nusrat and Harbig et al. (Nusrat et al., 2019) provide a broad overview of existing visualization techniques and tools for comparative genomics. This is the basis for the following discussion. There are generally three basic techniques for the visual comparison of genomes (gMSAs):

• comparison by alignment

• comparison by connecting conserved blocks

• comparison by using dot plots

The comparison using alignment based techniques is especially performed for small sequences or local analyses (Li et al., 2009; Carver et al., 2012; Yachdav et al., 2016).

When comparing the genomes with connected conserved blocks, the genomes (sequence intervals) are arranged on two or more axes and the synteny is displayed using color or line coding. For this, there are mainly two different types of layouts: linear and circular arrangements for comparing sequences. Examples for tools using linear layouts are Cinteny (Sinha and Meller, 2007) (using colors and lines) or Synteny Explorer (Bryan et al., 2017). Circular layouts are, for example, used by the tools MizBee (Meyer et al., 2009), Circos (Krzywinski et al., 2009), or Synteny Explorer (Bryan et al., 2017). With MizBee (Meyer et al., 2009), a tool is provided that supports analyses at the genome, the chromosome, and the block levels with circular and linear layouts. However, its line based approach is in some instances not suitable for a detailed comparisons on the genome level, since it tends to produce several intersections. Furthermore, most tools like MizBee (Meyer et al., 2009) are designed for the analysis of only two sequences.

In dot based approaches, the comparative sequence axes are arranged orthogonally and similarities are indicated by diagonal rows of dots. Tools using this approach are, for example,: Gepard (Krumsiek et al., 2007), EDGAR (Blom et al., 2016), Syn-Map2 (Haug-Baltzell et al., 2017), and iDotter (Gerighausen et al., 2017). Most of the tools only allow the comparative analyses of two sequences and do not scale well for the comparison of larger regions.

In the following, a brief overview of graph-related related work is given. We decided to use the Sugiyama framework (Sugiyama et al., 1981) as the basis to layout the gMSA graphs since it is by far the most common layout framework for directed graphs (Healy et al., 2013) and is highly adaptable.

Other graph-based approaches related to our problem setting can be found in the field of digital humanities. There, the comparison of different versions of a text is an important aspect. According to Schmidt et al. (Schmidt and Colomb, 2009), so-called *Text Variant Graphs* emphasize such overlapping textual structures. Jänicke et al. (Jänicke et al., 2014a; Jänicke et al., 2014b) developed *Sentence Alignment Flow*, a well readable layout algorithm for Text Variant Graphs. While their approach is similar to ours, these graphs are easier to visualize since texts always have the same reading direction, but this does not always have to be the case in gMSA graphs. Therefore, and since the sequences can be considerably longer and the differences are more complex in gMSA graphs, the development of a separate approach was necessary.

The following definitions are based on Chapter 7 of the “Handbook on Graph Drawing and Visualization” (Duncan et al., 2013). Let G=(V,E) be a *directed graph* with V being a finite set of *vertices* and E being a finite set of edges. An *edge* e∈E:e=(u,v) is an ordered pair of vertices, where u∈V is the *origin* and v∈V is the *destination* of the edge. Furthermore, e=(u,v) is an *incoming edge* of v and an *outgoing edge* of u . A *sink* is a vertex x∈V without outgoing edges and a *source* is a vertex y∈V without incoming edges. A sequence of distinct vertices $\left(v_{1},v_{2},\ldots ,v_{n}\right)$ is a *path* from $v_{1}$ to $v_{n}$ in G , if and only if n>1 and $\forall i\in N:1\leq i<n\to \left(v_{i},v_{i+1}\right)\in E$ and $\forall 1\leq i,j\leq n:i\neq j\to v_{i}\neq v_{j}$ . A *cycle* meets the same criteria as a path except that $v_{1}=v_{n}$ . The graph G=(V,E) is a *directed acyclic graph (DAG)*, if and only if there are no cycles in G . A *multi-graph* is a graph where the edges are multisets, which means two edges can have the same vertices as the origin and destination.

### 2.1 Data definition

**FIGURE 1:**
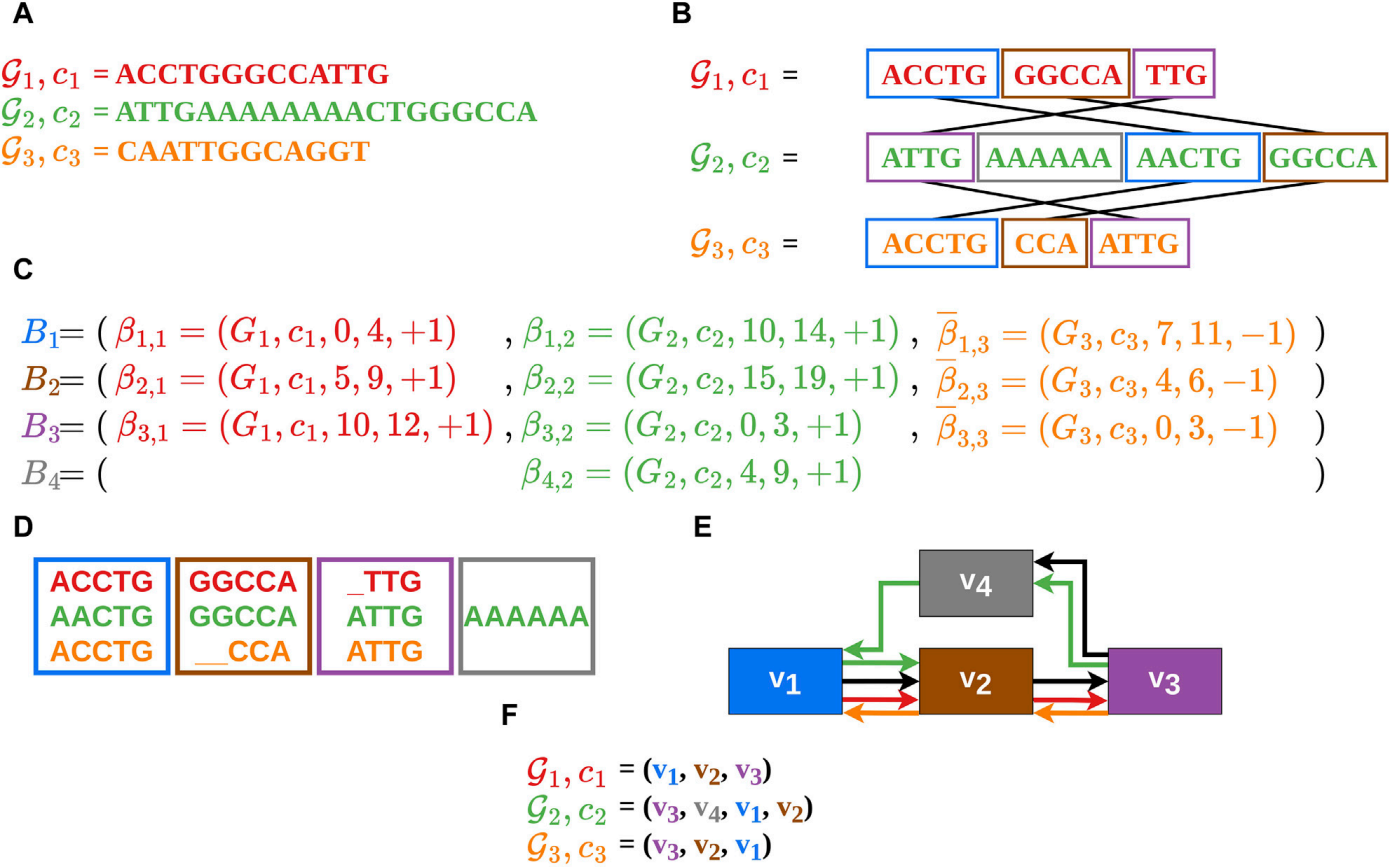
In this example the construction of an MSA A and its corresponding gMSA graph is shown. **(A)** The three nt-seqs each represent one contig of a genome to which a unique color is assigned: genome 1 - contig 1 (red), genome 2 - contig 2 (green), and genome 3 - contig three (orange). **(B)** The nt-seqs are split into sequence intervals and mapped onto the most similar sequence intervals of the other genomes using an alignment heuristic. It should be noted that $c_{3}$ of $G_{3}$ was completely transferred to the reverse complement in order to map it. **(C)** From this, the MSA A is built where the intervals are designated as $\beta_{k,l}=\left(G_{l},c_{l},i_{k,l},j_{k,l},+1\right)\in B_{k}$ and ${\bar{\beta }}_{k,l}=\left(G_{l},c_{l},i_{k,l},j_{k,l},-1\right)\in B_{k}$ . The four alignment blocks $B_{k}$ also have their own color coding. **(D)** Shows the alignment blocks on nt-seq level with the inserted gaps. The gray alignment block is a special case as there were no matching sequence intervals for $\beta_{4,2}$ in the other contigs and it is therefore alone in a block. **(E)** The MSA A can be illustrated as a gMSA graph. The vertices represent the alignment blocks and the edges represent the order of the sequence intervals in the contigs (recognizable by the color coding). The black edges represent a possible artificial reference order (supergenom). **(F)** The alignment block sequences/vertex sequences, which reflect the order of the sequence intervals in the contigs.

According to Wang et al. (Wang and Jiang, 1994), we define a *nucleotide sequence (nt-seq)* as an arbitrary, finite string $s\in \sum^{*}$ over the Alphabet ∑={A,C,G,T} which represents a single DNA strand (Figure 1A). The following formal definitions are strongly influenced by Gärtner et al. (Gärtner et al., 2018) as we use their data. Our initial data is a set of genome assemblies, which Gärtner et al. (Gärtner et al., 2018) referred to as assemblies, and we just call them *genomes*. For us, a genome is a set of nt-seqs that include substructures such as chromosomes, scaffolds, reftigs, contigs, and others. In the following, we will always refer to such substructures as *contigs*. Every sequence has a usual coordinate system defining the sequence positions. In addition, each sequence has a *direction*, since we are dealing with double-stranded DNA, that occurs either in the specified direction (σ=+1) or as a reversed complement (σ=−1) . With these specifications, (G,c,i,j,σ) describes a *sequence interval* from position i to j on contig c of genome G with a reading direction σ where i≤j . For the comparative analysis of genomes (usually more than 2), we use *multiple sequence alignments (MSA)*. An MSA A consists of *alignment blocks* each of which is composed of aligned sequence intervals (Figure 1C, D). Thus, an alignment block B∈A is defined as $B=\left\{\left(G_{u},c_{u},i_{u},j_{u},\sigma_{u}\right)|u\in \right.$ rows of $\left.B\right\}$ where the rows represent the contained sequence intervals. For each contig contained in the MSA A , a total order of the alignment blocks can be found that represents the original contig (Figure 1A) through the sequence intervals contained (Figure 1C). These alignment block sequences can be represented as a graph which we call *gMSA graph* (Figure 1E). Every vertex in the gMSA graph represents at least one alignment block. The order of the alignment blocks of a contig c is represented by the directed edges between the vertices. This allows tracing the contig (Figure 1A) in the gMSA and in the graph (Figure 1E). On this basis, Gärtner et al. (Gärtner et al., 2018) construct their common coordinate system (supergenome). It is possible to represent artificially created orders (like the supergenome by Gärtner et al. (Gärtner et al., 2018)) in the gMSA graph, which is an advantage over existing visualization techniques. Using such a gMSA graph, the structural differences and similarities of the contigs, and thus also the genomes, to be compared can be analyzed.

It should be noted, that a vertex in the gMSA graph can also represent a sequence of alignment blocks (*merged alignment blocks*) if no information is lost as a result. This is the case when two or more alignment blocks are traversed by only one contig or are traversed co-linearly in the same order by several contigs. In this manuscript, the terms alignment block (including merged alignment blocks) and vertex will be used as synonyms.

The *alignment block sequences* (also called *vertex sequences*), which reflect the contigs in the gMSA (Figure 1F), serve as the data basis for our visualization. Therefore, our input data consists of a list of *vertex sequences* S which represents the gMSA graph (multi-graph). One of the vertex sequences called *guide sequence* is selected as being the reference the other sequences are compared to. The remaining vertex sequences are compared to the guide sequence and are called *comparative sequences*. As already mentioned, the common coordinate system (supergenome) developed by Gärtner et al. (Gärtner et al., 2018) can also be used as a comparative sequence since it is also a vertex sequence. It is important to mention, that we only allow single hits in the alignments and therefore every sequence interval is uniquely aligned.

It should also be noted that each comparative sequence starts at an alignment block of the guide sequence and ends at another alignment block of the guide sequence. Between the start and the end alignment block of a comparative sequence there can be any number of alignment blocks, which do not necessarily belong to the guide sequence. These two conditions shall avoid loose ends of the comparative sequences, which are not shared with the guide sequence (as this should be in focus).

### 2.2 Design requirements

We had extensive discussions with our collaboration partners from the bioinformatics field about what is important and helpful to them for a comparative analysis of sequences. At the same time, we were inspired by well-known visualizations, such as classical genome browsers, and existing graph-based representations for alignment data, such as text variant graphs. With this, the following design requirements were derived:

1. The general reading direction of the graph is from left to right.
2. The guide sequence is represented linearly in the layout, i.e., at the same vertical position with a uniform direction.
3. The first vertex of the guide sequence is placed furthest to the left and the last one furthest to the right, i.e., at the smallest and largest horizontal position, respectively.
4. The comparative sequences are ordered by decreasing relevance, creating a genome order.
5. Sub-sequences of the comparative sequences are arranged above and below the guide sequence if they diverge from the guide sequence.
6. Contiguous sub-sequences of the comparative sequences are aligned horizontally, i.e., at the same vertical position, if possible.
7. If there is a deviation (e.g., an insertion) from the already processed sequences (predecessor in the genome order), these parts are placed between the corresponding vertices if possible.

The Requirements: 1–3 and 5–7 for the layout were highly inspired by the Text Variant Graphs by Jänicke et al. (Jänicke et al., 2014a; Jänicke et al., 2014b). The general reading direction of Requirement 1 (also in accordance with Requirement 3) describes especially the direction of the guide sequence in which no edge may be reversed (Requirement 2) and is therefore based on the familiar representation of genomic data in common genome browsers. Of course, back edges against the reading direction are generally possible in the comparative sequences. Requirement five supports the demonstration of similarities and differences between the sequences. Following Requirement 5, the space above and below the reference is used which creates a compact visualization, unlike classic genome browsers.

Not all comparative sequences can have an equally strong influence on the final layout. Therefore, the genome order of Requirement four is important and affects the graph layout in a strong manner, since the creation of the DAG (which is necessary for the Sugiyama framework) depends on it. This will be discussed further in Section 2.3.1. The length of the contiguous sub-sequences of Requirement six also depends on the genome order (Requirement 4). The same holds for the deviations (Requirement 7).

We impose additional constraints (adapted from Healy and Nikolov (Healy et al., 2013)):

1. Edges should point in a uniform direction,
2. Short edges are preferred over long edges,
3. The vertices should be uniformly distributed in the drawing space,
4. Edge crossings should be avoided, and
5. Straight edges should be preferred

These constraints are frequently imposed in general onto the layout of directed graphs (e.g., by the Sugiyama framework).

### 2.3 Framework

Our layout algorithm for gMSA graphs is based on the Sugiyama framework (Sugiyama et al., 1981; Healy et al., 2013) (Steps 2–4). While the original framework applies to DAGs only, it was extended to general directed graphs by adding one step at the beginning (Step 1) and at the end (Step 5) of the original framework. In addition of being cyclic, our graphs also contain multiple edges between vertices. Therefore, an additional step for routing these edges was added to the framework (Step 6). At the end, the final layout is computed (Step 7). Overall, we obtain the following extended framework:

1. Cycle removal by reversing edges (Section 2.3.1)
2. Layer assignment (Section 2.3.2)
3. Vertex ordering (Section 2.3.3)
4. Orthogonal coordinate assignment (Section 2.3.4)
5. Reversing the edges reversed in Step 1 (Section 2.3.5)
6. Routing the edges between vertices (Section 2.3.6)
7. Computing the final drawing (Section 2.3.7)

For each step, we describe, which algorithms were used for performing the respective step. Besides taking algorithms from literature, we also adapted known algorithms and created new ones for meeting the requirements (Section 2.2). A data flow diagram of the described framework is shown in the Supplementary Section S1.

The Sugiyama framework has the following additional properties:

1. The algorithm produces a layered graph layout, i.e., every vertex is assigned to exactly one layer.
2. There are no edges between two vertices of the same layer.
3. Crossings of the edges are minimized.
4. Crossings between edges and vertices are avoided.

It is generally assumed that edge crossings impede the readability of graphs and create visual clutter. This is mitigated by reducing the number of edge crossings as reflected by Property 3.

As the requirements partially contradict the properties of the Sugiyama Algorithm such as, e.g., Requirement five and Property 3, it is necessary to consider which is more important for the resulting visualization. In the example mentioned, the straight arrangement of genome parts (Requirement 5) is more important for the visual comprehension than the minimization of the edge crossings (Property 3), which will be reflected by our algorithms.

#### 2.3.1 Cycle removal

Although each alignment block sequence (contig) is cycle-free by itself, there may (and frequently will) be cycles in a gMSA graph due to the sum of the contigs. The simplest example for this is if contig one first visits alignment block one and then alignment block two in its sequence and this is reversed for contig 2. Thus, a preparation step is necessary to create a proper DAG which can be processed by the Sugiyama framework. Therefore, the orientation of some of the edges of the original graph has to be reversed. This step is very important because the edge selection strongly influences the layering and with this the resulting layout.

**FIGURE 2:**
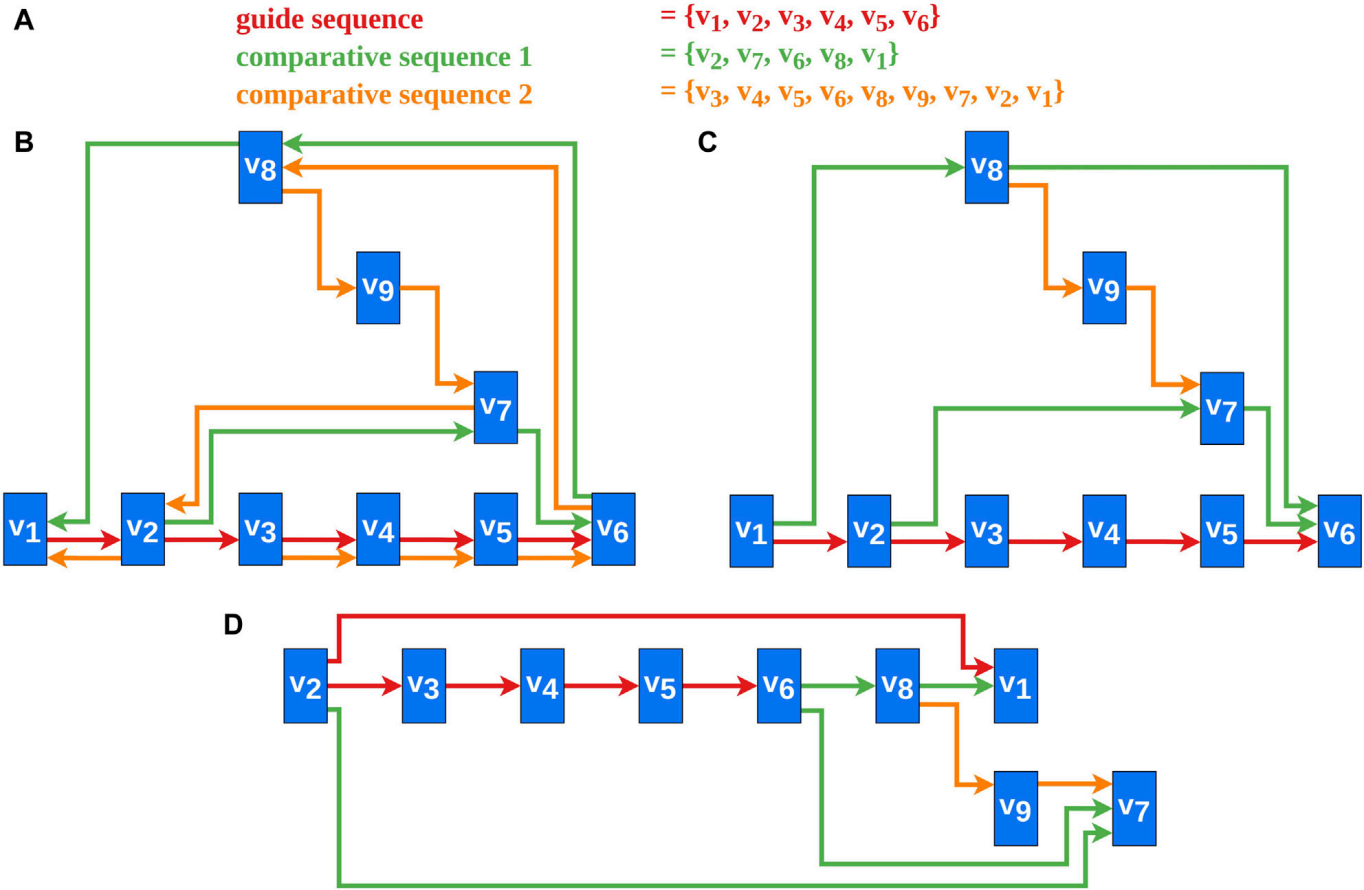
An example of our new cycle removal algorithm and the results for a greedy heuristic (Eades et al., 1993) are shown. **(A)** The vertex sequences of the selected sequences. This is the data basis for the graph created in this example. **(B)** A possible final graph representation of the data from **(A)**. **(C)** A DAG created by applying Algorithm one to the data from **(A)**. The bypath $\left(v_{6},v_{8},v_{1}\right)$ was reversed. This is important to fulfill the design requirements (Section X). **(D)** A possible DAG created by the greedy heuristic by Eades et al. (Eades et al., 1993). The Requirements 2,3, and seven are violated in this result. $v_{1}$ being a sink is especially problematic for the resulting graph layout.

Several heuristics address the problem of reversing a minimal number of edges for transforming a directed graph containing cycles into a DAG, as this problem is NP-complete (Di Battista et al., 1999; Healy et al., 2013). The heuristics by Berger and Shor (Berger and Shor, 1990) and Eades et al. (Eades et al., 1993) use approaches based on linear ordering. Another approach by Gansner et al. (Gansner et al., 1993) uses a depth-first traversal of strongly connected components to solve this problem. According to them (Gansner et al., 1993), the heuristic reverses edges whose direction against the flow is natural. The heuristic by Demetrescu and Finocchi (Demetrescu and Finocchi, 2003) solves the problem for weighted directed graphs. For us, however, the comparative aspect of the vertex sequences, by meeting our requirements (Section 2.2), is more important than the number of reversed edges. For example, the greedy heuristic by Eades et al. (Eades et al., 1993) was tested and even adapted without success (Figure 2D). This and similar techniques potentially produce sinks or sources in the DAG and therefore Requirements 2, 3, and seven are potentially violated, as sinks and sources would be placed furthest to the right or to the left (Figure 2D). Therefore, a new approach for edge reversal was developed that adheres to these requirements. An example of a DAG created by the new algorithm is shown in Figure 2C.

The genome order (Requirement 4) has an important significance for this algorithm and thereby for all the subsequent steps. The higher the priority of a comparative sequence in the genome order, the higher the influence on the DAG and thus on the final layout.

Before describing our algorithm, we introduce the following definitions. A *vertex sequence* $S_{i}$ is a path. The input of the algorithm is the *list of vertex sequences* $S=\left(S_{1}=GS,\ldots ,S_{n}\right)$ where the first vertex sequence represents the guide sequence (GS) and the remaining vertex sequences represent the comparative sequences. As already mentioned, the list of vertex sequences is a representation of the gMSA graph and is ordered by importance (genome order).

We introduce the notion of a *bypath*. Let $V_{p}=\left\{v\in V|v\text{already processed}\right\}$ be the set of *already processed vertices* of the gMSA graph during the algorithm. Further, let

$$\begin{array}{cccc} \sigma : & Vp & \to & 2^{Vp}\\ & v & \mapsto & \sigma_{v}=\left\{w\in Vp|\exists path\left(v,...,w\right)\right\} \end{array}$$ (1)

be a *successor function* (see Equation 1), mapping each vertex v to its successors, i.e., the set of all vertices $\sigma_{v}$ such that there is a path from v to $w\in \sigma_{v}$ . Similarly, let

$$\begin{array}{cccc} \tau : & Vp & \to & 2^{Vp}\\ & v & \mapsto & \tau_{v}=\left\{u\in Vp|\exists path\left(u,...,v\right)\right\} \end{array}$$ (2)

be a *predecessor function* (see Equation 2), mapping each vertex v to its predecessors, i.e., the set of all vertices $\tau_{v}$ such that there is a path from $u\in \tau_{v}$ to v .

A sub-path $\left(v_{1},v_{2},\ldots ,v_{n}\right)\subseteq S_{i}$ with n>1 , $v_{1},v_{n}\in V_{p}$ , and $\forall i\in N,1<i<n:v_{i}\notin V_{p}$ is called a *bypath*. In other words, a bypath is associated to a vertex sequence and starts and ends at an already processed vertex but may have only unprocessed vertices in between.

For layout purposes, the set of edges $E_{\mathrm{DAG}}$ resulting from the cycle removal algorithm will contain only one edge between two vertices, while in the gMSA graph multiple edges between two vertices are possible. Note, that having both edges (u,v) and (v,u) is not possible in a DAG. To associate the edges of the DAG to the edges of the original gMSA graph that finally need to be drawn, we introduce the following concepts. The *direction tuple set* D (see Equation 3) consists of tuples (d,S) where an *edge direction* d∈{forward,backward} is combined with a vertex sequence S∈S :

$$\begin{array}{c} D=\left\{forward,backward\right\}\times S \end{array}$$ (3)

For $S=\left(v_{1},\ldots ,v_{n}\right)$ , let $E_{S}=\left\{\left(v_{i},v_{i+1}\right)|1\leq i<n\right\}$ . Further, let

$$\begin{array}{ccccc} \epsilon : & E_{\mathrm{DAG}} & \to & D\\ & eDAG=\left(u,v\right) & \mapsto & \epsilon_{e}= & \left\{\left(forward,S\right)\;\;|\left(u,v\right)\in E_{S}\right\}\\ & & & & \cup \;\left\{\left(backward,S\right)|\left(v,u\right)\in E_{S}\right\} \end{array}$$ (4)

be a function (see Equation 4), mapping an edge from the DAG eDAG∈EDAG to a set of direction tuples $\epsilon_{e}\subseteq D$ .

The pseudo code of the algorithms described in this section is provided in in the Supplementary Section S2.1. The original graph is represented by the list of vertex sequences S (Figure 2A) and may contain cycles. The graph is transformed into a DAG by first processing the guide sequence, and then processing the remaining vertex sequences in the order of the list S (genome order) satisfying Requirement 4. The remaining vertex sequences are split into bypaths based on the already processed vertices $V_{p}$ . If $v_{n}\in \sigma_{v_{0}}$ , that is, if the last vertex of the bypath BP is a successor of the first vertex of the bypath, then the bypath BP is added in its original direction. Otherwise, the bypath BP is added in the opposite direction. Reversing the edges of the complete bypath with $v_{0}\in \sigma_{v_{n}}$ avoids creating potential additional sinks or sources and is essential to fulfill the Requirement 2, 3, and 7 (Figure 2D).

Depending on which comparative sequences are processed first, the decomposition of these vertex sequences in the bypaths will be strongly affected. This also influences the amount of reversed edges, the created DAG, and therefore the subsequent steps of the Sugiyama framework. Since the similarities in vertex sequences between phylogenetically closely related genomes should be large, the priority of a comparative sequence should be higher whenever its relation to the guide sequence is higher. This promotes the visual comparability of the genomes in the gMSA graph layout. It could therefore be advantageous to sort the comparative sequences according to their phylogenetic proximity to the guide sequence, creating a genome order (Requirement 4).

The edges of the guide sequence are always added in their original direction to fulfill Requirement 2. For a given bypath BP , all of its edges point either in the same direction as the ones of the guide sequence or in the opposite direction. To obtain a uniform direction for later steps, in the first case, the edges of the bypath BP are added in their original direction, while in the second case, the edges of the bypath BP are reversed before being added.

First, between all adjacent vertex pairs u and v of the guide sequence and the bypaths, edges e=(u,v) or $e_{R}=\left(v,u\right)$ are created. Next, the nodes u and v are added to $V_{p}$ . Moreover, the edge e , or the edge $e_{R}$ is added to $E_{\mathrm{DAG}}$ . Further, the successor function σ is updated with the edge e=(u,v) in two steps:

1. v and all successors of v are inserted into $\sigma_{u}$ , if not already present: $\sigma_{u}\leftarrow \sigma_{u}\cup \left\{v\right\}\cup \sigma_{v}$

2. For all predecessors $w\in \tau_{u}$ , all successors of u are inserted into $\sigma_{w}$ , if $v\notin \sigma_{w}$ : $\sigma_{w}\leftarrow \sigma_{w}\cup \sigma_{u}$

If the bypath needs to be reversed, the reversed edge $e_{R}=\left(v,u\right)$ is used instead and all u and v are interchanged. In the same method, the predecessor function τ is updated with the edge e=(u,v) in these two steps:

1. u and all predecessors of u are inserted into $\tau_{v}$ , if not already present: $\tau_{v}\leftarrow \tau_{v}\cup \left\{u\right\}\cup \tau_{u}$

2. For all successors $w\in \sigma_{v}$ , all predecessor of v are inserted into $\tau_{w}$ , if $u\notin \tau_{w}$ : $\tau_{w}\leftarrow \tau_{w}\cup \tau_{v}$

Again, if the bypath needs to be reversed, the reversed edge $e_{R}=\left(v,u\right)$ is used instead and all u and v are interchanged. These four steps are always performed for the guide sequence, while they are only performed for any bypath if the edge e or the edge $e_{R}$ are not already part of the DAG. Consequently, there will be at most one edge between two vertices in the DAG at any time.

The direction tuple set $\epsilon_{e}$ or $\epsilon_{e_{R}}$ (depending on not reversing or reversing the bypath) of the function ϵ is always updated. Therefore, a tuple consisting of the direction with respect to the DAG (‘forward’ or ‘backward’) and the currently processed vertex sequence S is created and added to $\epsilon_{e}$ or $\epsilon_{e_{R}}$ . This information is needed and used to create the final layout, such that all edges can then be drawn in their original direction in the final layout.

In Supplementary Section S2.2 an example is provided showing the results for the sets $V_{p}$ and $E_{\mathrm{DAG}}$ as well as for the mappings σ , τ , and ϵ , when adding each of the sequences shown in Figure 2A. Furthermore, a differentiation from an ear decomposition and a complexity analysis are included in the Supplementary Section S2.3, 2.4.

The result of this step is a DAG with only one source (the first vertex of the guide sequence) and only one sink (the last vertex of the guide sequence) (Figure 2C). This is necessary but not sufficient for fulfilling the Requirements 1, 2, 3, and 7. These requirements are only completely met after the layering step (Section 2.3.2). Besides, this step is the foundation for fulfilling Requirement six since the length of the sub-sequences is determined by the bypaths. Requirement four is essential for the resulting layout since with every change in the genome order, the DAG created and thus the final layout could be completely different. Finally, the mapping of the multiple edge function ϵ produced here will be used in subsequent steps.

#### 2.3.2 Layer assignment

For assigning the vertices to layers, quite some algorithms meeting different requirements exist (Healy et al., 2013). For the layer assignment, we use the longest-path algorithm without any adaption. A more detailed explanation of the algorithm with associated pseudo code can be found in the Supplementary Section S3.

It is worth emphasizing that our cycle removal algorithm from Section 2.3.1 paired with the properties of the longest-path algorithm satisfies the Requirements 1, 2, 3, and 7, since the guide sequence is the longest path in the graph. The resulting layers represent the *horizontal position (x-coordinate)* of the vertices in the final layout.

For the subsequent steps of the framework, a proper layering is required, which can be obtained from the results of the longest path algorithm as follows (Healy et al., 2013). Let $L=\left\{L_{1},L_{2},\ldots ,L_{h}\right\}$ be a partition of V into h≥1 subsets such that $\forall u\in L_{i}\wedge v\in L_{j}:\left(u,v\right)\in E_{\mathrm{DAG}}\to i<j$ . Then, L is called a *layering* of G and the subsets $L_{1},\ldots ,L_{h}$ are called the *layers*. In a layering, all edges of the DAG point from a vertex in a lower layer to one in a higher layer, and in our case they will be drawn in the layout from left to right.

Let l(u)=i be the index of the Layer $L_{i}$ which contains the vertex u∈V . If the *span* s(e)=l(v)−l(u)>1 of an edge e=(u,v) , the edge is called *long edge* and therefore traverses more than one layer. In contrast to this, edges with a span s(e)=1 are called *tight edges*. A layering L without long edges is called *proper* and can be obtained as follows: all long edges are replaced by s(e) many tight dummy edges by creating s(e)−1 dummy vertices and placing them in the layers that are traversed by the long edge. In other words, the path of the long edge is replaced by the path of the dummy edges with the dummy vertices in between.

An edge between two dummy vertices is called *inner edge* and all other edges are called *outer edge*. All dummy vertices of a properly layered DAG are added to the set of dummy vertices $V_{D}$ and to the set of vertices of the DAG $V_{\mathrm{DAG}}=V\cup V_{D}$ while the dummy edges are added to the set of dummy edges $E_{D}$ and to the set of edges of the DAG $E_{\mathrm{DAG}}=E_{\mathrm{DAG}}\cup E_{D}$ . Further, every replaced long edge is removed from the set of edges of the DAG. In the function ϵ , all dummy edges of a replaced long edge e are mapped to the same set as the replaced long edge $\epsilon_{e}$ . The mapping of e to $\epsilon_{e}$ in ϵ is removed.

In the following, the notion $G_{\mathrm{DAG}}=\left(V_{\mathrm{DAG}},E_{\mathrm{DAG}},L\right)$ is used for the properly layered DAG.

#### 2.3.3 Vertex ordering

An important step of the Sugiyama framework is the reduction of edge-edge crossings (edge crossings) to improve the readability and to avoid visual clutter. There are three different crossing types, depending on the involved number of inner edges:

• **Type 0:** two outer edges are crossing each other

• **Type 1:** an inner and an outer edge are crossing each other

• **Type 2:** two inner edges are crossing each other

Especially the Type 2 conflicts should be avoided because they produce crossings of long edges, which are hard to follow even without crossings. Moreover, Type 2 conflicts can always be avoided and their absence is a precondition for some algorithms computing the orthogonal coordinates.

To minimize the number of those crossings, the order of the vertices within the layers is changed. We adapted the global k-level crossing reduction heuristic called *global sifting* that was introduced by Bachmaier et al. (Bachmaier et al., 2010) such that the Requirements 2, 5, and six are fulfilled.

The following formal explanations are based on Bachmaier et al. (Bachmaier et al., 2010). Let the graph $G_{\mathrm{DAG}}=\left(V_{\mathrm{DAG}},E_{\mathrm{DAG}},L\right)$ be an *ordered* properly layered graph with its vertices in each layer being ordered top down (vertically).

**FIGURE 3:**
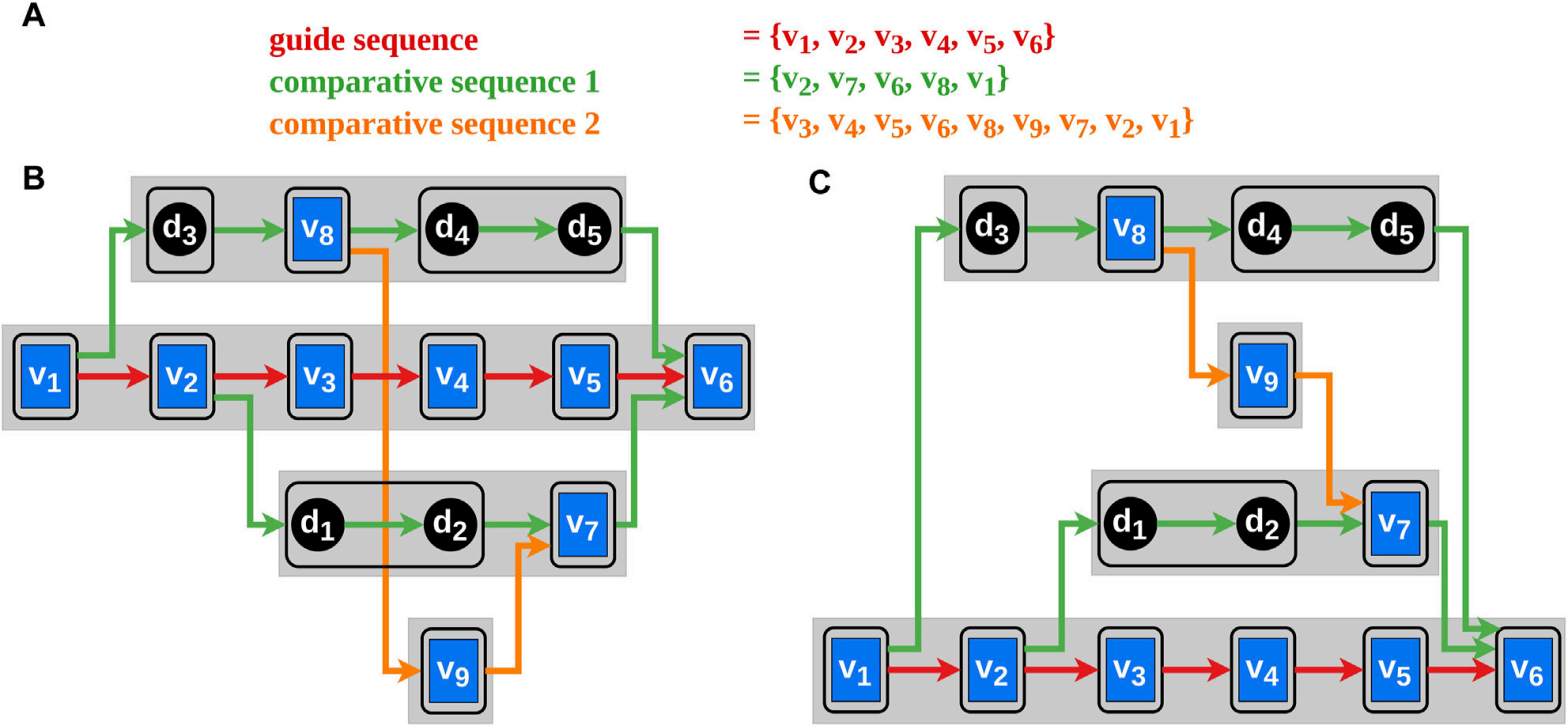
The blue rectangles in the graphs represent the vertices and the black circles the dummy vertices. The edges between them indicate the order of the selected vertex sequences corresponding to their color. The black frames around single vertices or sequences of dummy vertices illustrate blocks. Block-sets are represented by the gray rectangles. Each block-set has a unique vertical position in the block-set order. **(A)** The vertex order of the selected genomes. **(B)** A possible initial block-set order with avoidable edge intersections. **(C)** A possible block-set order after applying our algorithm.

A *block* B is either a single vertex v∈V or a maximum path of adjacent dummy vertices $\left(v_{1},\ldots ,v_{n}\right),v_{i}\in V_{D},\left(v_{i},v_{i+1}\right)\in E_{D}$ comprising the inner edges of a long edge. Thus, every vertex v∈V and every dummy vertex $v_{D}\in V_{D}$ is assigned to a block B , which is retrievable by block(v)=B and $block\left(v_{D}\right)=B$ , respectively (Figure 3).

We introduce the term *block-set* BS , which is a sequence of consecutive blocks of the inner vertices of a bypath (Section 2.3.1), including the dummy vertices of the long edges. Consequently, every bypath defines a block-set (Figure 3), except for bypaths of length 2, which just consist of a short edge without vertices.

Let BS be an arbitrarily ordered list of all block-sets and let the current position of each block-set in this ordering be assigned by η:BS→{0,…,|BS|−1} . The blocks in a block-set are sorted increasingly from lowest to highest layer. Like in the original heuristic, every block B is placed at a unique position π(B) that depends on the position of the corresponding block-set BS in the list BS and the position of the block B in the block-set BS .

The idea of the heuristic proposed by Bachmaier et al. (Bachmaier et al., 2010) is to place a block at every position (every block has a unique vertical position) thus finding the optimal position of the block with minimal edge intersections. This is done successively for every block and repeated several times (according to the original heuristic ten sifting rounds suffice). Using the original heuristic, the Requirements two and 6 may be violated by placing the blocks of a block-set far apart concerning their vertical position so that they cannot be horizontally aligned during the next step of the Sugiyama framework. In our heuristic, block-sets, which consist ordinarily of a sequence of blocks, are shifted instead of the blocks. By doing this, the connected blocks of the bypaths can be placed horizontally aligned thus complying with the Requirements two and six (illustrated in Figure 3). The result of this algorithm is an order of the block-sets BS , with a heuristically calculated minimum of edge crossings and an avoidance of type 2 conflicts. A detailed description of the adapted algorithm, including pseudo code, and a complexity analysis can be found in the Supplementary Section S4.

#### 2.3.4 Assignment of the orthogonal coordinate

For each vertex $v\in V_{\mathrm{DAG}}$ , its relative horizontal position ( x -coordinate) is obtained from its layer determined previously (Section 2.3.2), i.e., x(v)=l(v) . Moreover, the order of all vertices $v\in V_{\mathrm{DAG}}$ within the same layer l(v) was fixed (Section 2.3.3). Now, the vertical position ( y -coordinate) that is orthogonal to the layers will be determined for each vertex $v\in V_{\mathrm{DAG}}$ while retaining the order in this direction for each layer.

According to Healy and Nikolov (Healy et al., 2013), straight edges (especially for long edges) and vertices that are centered with respect to their neighbors are aesthetically desirable in this step. A standard algorithm for this purpose is the approach by Brandes and Köpf (Brandes et al., 2002), which is linear in time in the number of vertices and edges, and allows at most two bends per edge. Unfortunately, this approach might violate some of our requirements. For all vertices of a block-set, the vertical positions have to be the same to fulfill the Requirements 2, 5, and 6. This is not always the case when using the algorithm by Brandes and Köpf (Brandes et al., 2002). Therefore, a new approach was developed where the vertical positions of the block-sets and thus also the vertical positions of the vertices are determined. This algorithm also guarantees at most two bends per edge in the final layout like the algorithm by Brandes and Köpf (Brandes et al., 2002).

The output of the previous step (Section 2.3.3) was a sorted block-set list BS where the positions of the block-sets in the list are equivalent to unique vertical positions, i.e., no two block-sets have the same vertical position. The current step aims at minimizing the vertical space needed by the graph without creating additional edge crossings. At the same time, it is attempted to shorten the vertical length of the edges between the block-sets.

In two steps, all block-sets are placed as closely as possible to the guide sequence without creating intersections between the block-sets. During the first step, the block-sets before the guide sequence in BS and during the second step, the block-sets after the guide sequence are processed, respectively. Only the first step will be discussed, as the second step is handled symmetrically.

As a prerequisite, the index of the guide sequence $index_{GS}$ has to be known. The sorted block-set list BS is processed backwards from the block-set just before the guide sequence with $index=index_{GS}-1$ to the first block-set in the list with index=0 . The current block-set BS is thereby stacked at the next free vertical position above the guide sequence, where no intersections with other block-sets occur.

This step defines the relative vertical position for all vertices (block-sets) and fulfills the Requirements 2, 5, and 6. Improving the vertical position of an aesthetically unpleasing special case and a complexity analysis of this approach are discussed in the Supplementary Section S5.

#### 2.3.5 Preparing the graph to be drawn

The last step of the Sugiyama framework, putting back the edges reversed in Section 2.3.1 in their original direction, is not necessary in this framework because the edges of the DAG are mapped by the multiple edge function ϵ to the edges of the input graph with their original direction. Instead, a preparation step handling dummy paths that represent the long edges $E_{L}$ is performed. Thereby, the number of dummy vertices and the number of dummy edges are reduced. A detailed explanation of this step is provided in the Supplementary Section S6.

#### 2.3.6 Edge routing

The Sugiyama framework was created for directed acyclic graphs with *single* edges between two nodes. The gMSA graph, however, is a multi-graph with potentially multiple edges between two vertices. Therefore, a routing algorithm for multiple edges is proposed, which is applied after the positioning of the vertices using the Sugiyama framework.

The multiple edges of G=(V,E) are represented by the multiple edge function ϵ . Every edge $e\in E_{\mathrm{DAG}}$ is mapped to a set of direction tuples where every tuple represents an edge of the layout graph with an associated vertex sequence S∈S and the edge direction (“forward” or “backward”) relative to the reading direction. The direction tuples of ϵ are used as the input of this step and called edges in the following unless explicitly stated otherwise.

**FIGURE 4:**
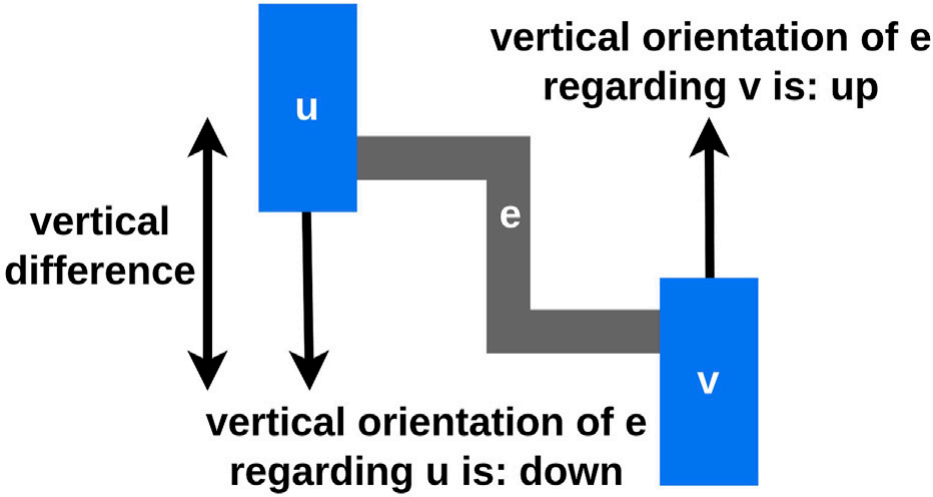
Let e be the edge between the two vertices u and v , where the direction of e is disregarded. The y -position of v is below the y -position of u , therefore the vertical orientation of e regarding u is downward, while the vertical orientation of e regarding v is upward.

Let e be the edge between the vertices u and v with the edge direction not being important (Figure 4). The *vertical orientation of the edge* e , regarding u is *downward (down)*, if the y -position of v in the layout is below the y -position of u . Consequently, the vertical orientation of e regarding u is *upward (up)*, if the y -position of v in the layout is above the y -position of u . In case both vertices are on the same y -position, the vertical orientation is *straight*. The *vertical difference* between u and v is the absolute value of the y -position difference between those two vertices. Moreover, the *inter layer space* describes the area between two adjacent layers.

**FIGURE 5:**
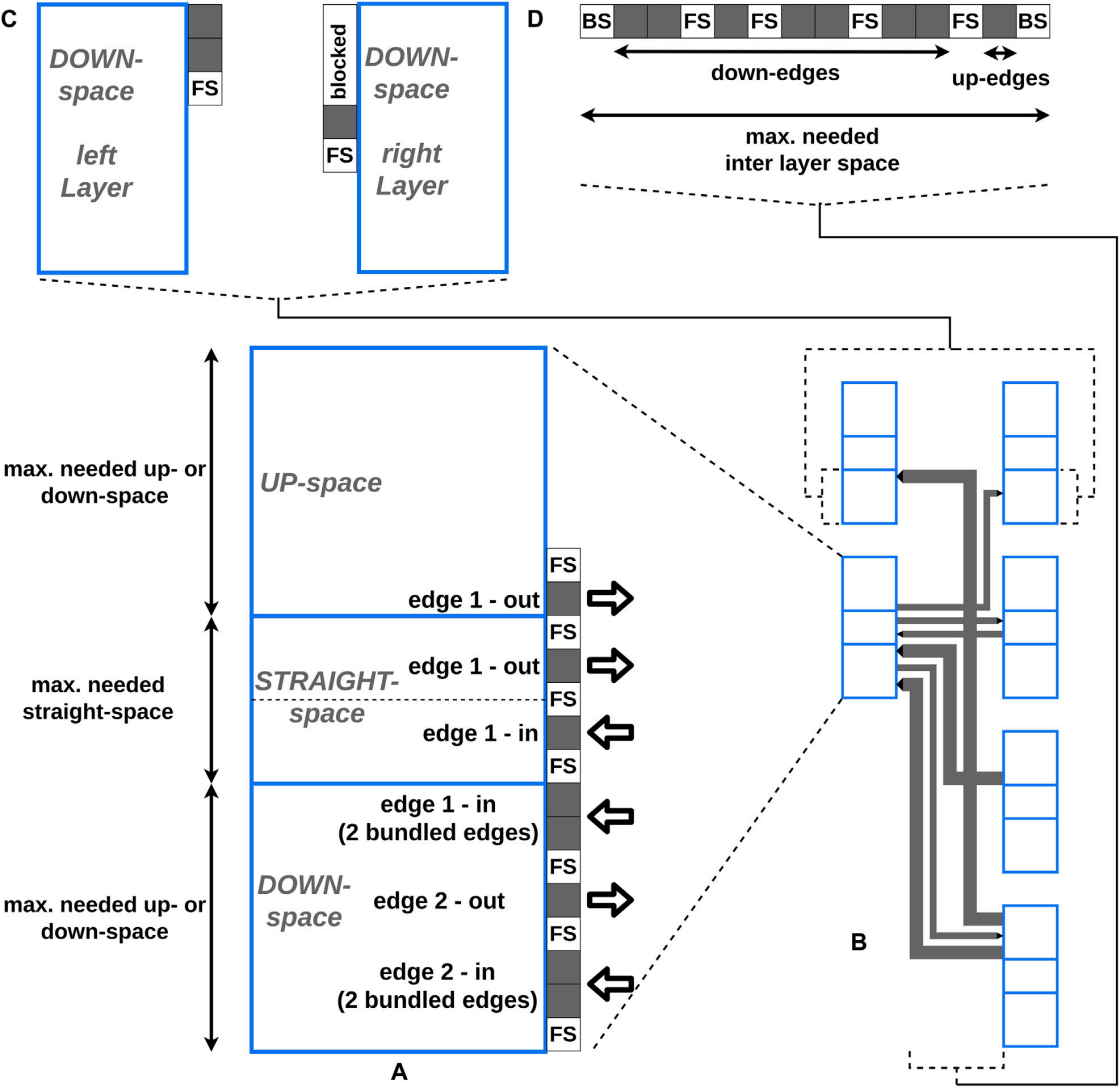
The blue boxes represent the vertices, whereby every vertex consists of three areas: the up-, the straight-, and the down-space. The dotted line in the straight-edge space of **(A)** illustrates the vertical center of the vertex. The gray boxes represent the edge positions. “FS” is the abbreviation for free space and represents the amount of free space between two edges. “BS” stands for border space and represents the amount of free space between the border of a vertex and the vertical line of an edge. **(A)** A detailed view of placing edges at the right border of a vertex is shown. **(B)** The final edge layout between two layers created by our approach is shown. One of the vertices is shown in **(A)** in detail. **(C)** The detailed view of the down-space of two opposite vertices from **(B)** is shown. To avoid (partial) overlaps, first the down-edges from the left vertex and afterwards the down-edges from the right vertex are placed. The space used for the down-edges of the left vertex is blocked and can not be used at the right vertex. **(D)** The detailed view of the horizontal layering of the vertical lines of all edges from **(B)** in the inter layer space is shown. Every vertical line of an edge has a unique horizontal position in the layout to avoid additional intersections.

Our requirements for routing the edges are the avoidance of additional edge intersections and a compact edge packing, which still supports readability (Figure 5B). To achieve these requirements, we adhere to the following conventions and impose the following constraints:

• An orthogonal edge layout consisting of horizontal and vertical lines is used. A vertical line is only needed, if the vertical orientation of the edge is either up or down.

• Edges start and end at the right and left borders of the vertices, only.

• Edges starting and ending at the same vertices with the same edge direction are bundled into one edge. The line width of a bundled edge represents the number of the edges bundled. This allows one edge in the DAG be represented by at most two bundled edges in the final layout (forward and/or backward).

• The vertical and horizontal lines of forward and backward edges with the same start and end vertex are placed adjacent to each other, respectively.

• The contact point of an edge at a vertex depends on the vertical orientation of the edge and is independent of the edge direction. Up-edges are placed at the upper part, straight-edges are placed in the middle, and down-edges are placed at the lower part of a vertex, respectively (Figure 5A).

• Between two adjacent edges, the same amount of free space “FS” is used as placeholder.

• Every vertical line of an edge has a unique horizontal position in the layout to avoid additional intersections (Figure 5D).

To determine the vertex-edge connection points and the horizontal positions of the vertical lines of an edge, four steps are performed:

1. Preprocessing
2. Calculating relative edge-vertex connection coordinates
3. Horizontal edge layering in the inter layer space
4. Determining the space parameter

The first step is a preparation step where the left and the right sides of every vertex are processed consecutively. During the second step, the relative coordinates of the edge-vertex connections are calculated. During the third step, the horizontal positions of the vertical lines of the edges in the inter layer space are determined. During the fourth step, the two space parameters *needed vertex height* and *needed inter layer space*, which are necessary for the final drawing (Section 2.3.7), are calculated. A detailed description of the algorithms and a complexity analysis are provided in the Supplementary Section S7. With the chosen orthogonal layout, additional edge intersections are avoided and a compact edge packing that still supports readability was produced.

#### 2.3.7 Final drawing

**FIGURE 6:**
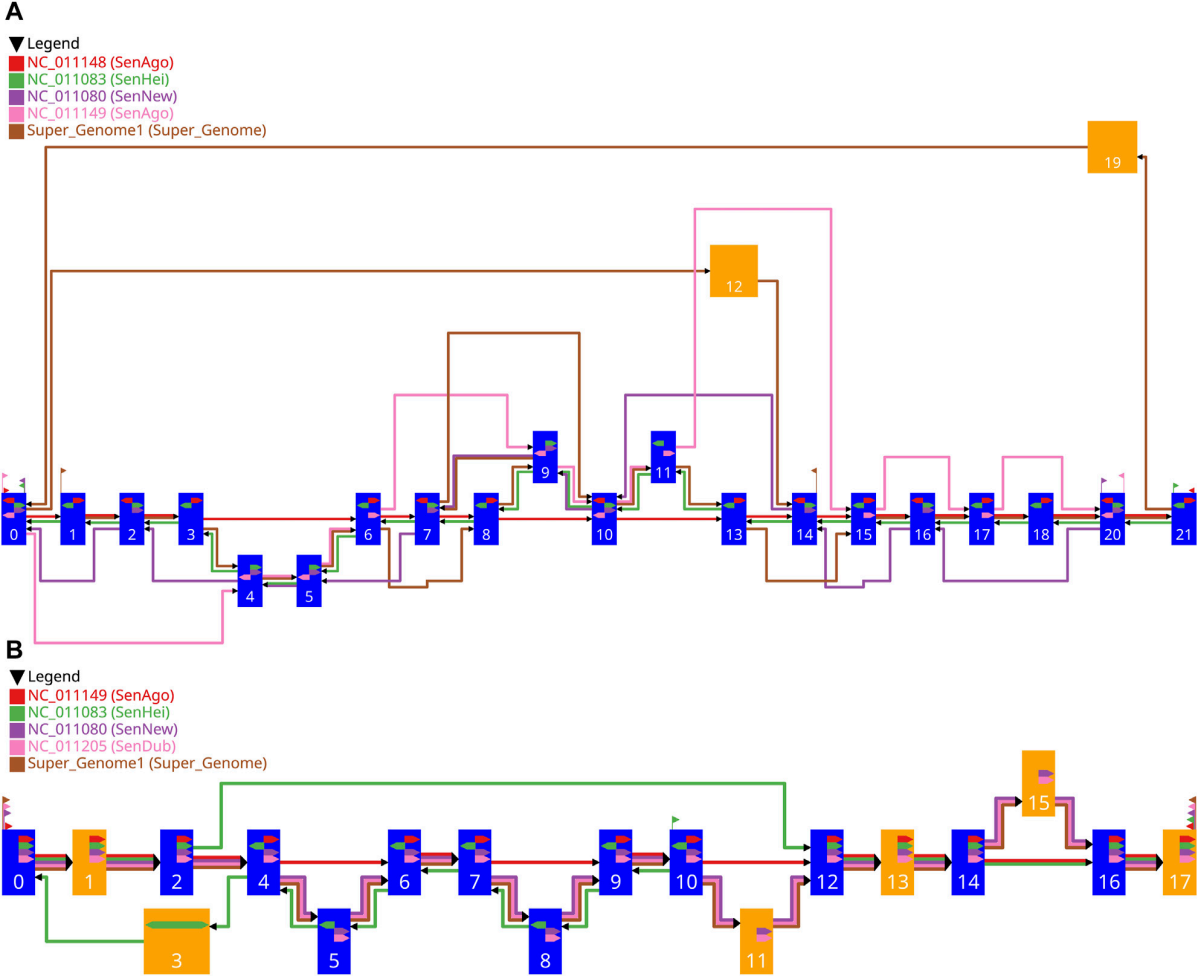
Two examples of the drawing of a gMSA graph generated with our workflow. The settings used for the graph in **(A)** are shown in Table 1 and for the graph in **(B)** in Table 2. The blue vertices represent single alignment blocks and the orange vertices represent merged alignment blocks. The width of a vertex depends on the length of the included alignment block(s) and is between pre-defined limits. The colored edges represent the different sequences, whereby the red edges represent the guide sequence and the other colors the comparative sequences. The directional glyphs (colored arrows) within the vertices indicate the reading direction for the individual sub-sequences aligned. Since there are no aligned sub-sequences for the supergenome, there are also no directional glyphs. The start and end glyphs (triangular flag) illustrate for each contig which vertex is the first one or the last one, respectively.

In this final step, the gMSA graph is drawn. First, every vertex v∈V of the graph is drawn depending on the relative x- and y-coordinate. Then, the relative coordinates of every edge e∈E (represented by ϵ ) are converted into the final coordinates. Two examples are shown in Figure 6.

The vertices are drawn as rectangles. Blue vertices represent single alignment blocks and the orange vertices represent merged alignment blocks. Every vertex has the same height, which is at least the *needed vertex height*, which was calculated before (Section 2.3.6). The width of a vertex depends on the length of the included alignment block(s) and is between pre-defined limits. The vertices are positioned on a grid, with the block-set position reflecting the vertical position and the layer affiliation reflecting the horizontal position. Therefore, the final vertical position of a vertex depends on the vertical position of its block-set. The vertical free space between two vertically adjacent vertices is always the same. The final horizontal position of a vertex depends on the assigned layer. Since the vertices in a layer can be of different width, the vertices of a layer are vertically aligned with their horizontal midpoint. Consequently, the width of the widest vertex in the graph defines the needed width for every layer. The horizontal free space between two layers, where the edges are located, is always the same and is at least the *needed inter layer space*, which was calculated before (Section 2.3.6). With this information all vertices can be drawn.

Having fixed the final coordinates of the vertices, the relative coordinates of the edges (Section 2.3.6) can be transformed into final coordinates and drawn. Each edge is drawn individually, including the individual edges in a bundled edge. This makes it easier to follow the route of the individual vertex sequences from S , if each vertex sequence (contig) is assigned an individual color.

Additionally, the directional glyphs (colored arrows) within the nodes indicate the reading direction for the individual sub-sequences aligned. The color codes the affiliation to the contig, a glyph pointing to the right (reading direction in the graph) means a positive direction of the aligned sub-sequence, and a glyph pointing to the left means a negative direction. For merged vertices, it is possible that a glyph pointing in both directions indicates that there are subsequences in both directions in the contained alignment blocks.

The start and end glyphs (triangular flag) illustrate for each contig which vertex is the first or last, respectively. The color codes the affiliation to the contig, a glyph pointing to the right (reading direction in the graph) means that the sequence begins here, and a glyph pointing to the left indicates the end of a sequence.

The final result is a heuristically generated drawing of the input gMSA graph where all requirements (Section 2.2) are fulfilled. A complexity analysis of this step is discussed in the Supplementary Section S8.

## 3 Results

In this section we discuss two examples of a gMSA graph layout that were created with our new framework. The examples are sub-graphs of the data set described in the Supplementary Section S9. With the graph layout generated, the similarities and the differences between the guide sequence and the comparative sequences are visually highlighted. Therefore, all requirements of Section 2.2 are fulfilled.

### 3.1 Example 1

In the first example, four comparative sequences are compared with a guide sequence. The genome order chosen as well as the coloring assigned to the sequences and the selected range are presented in Table 1.

**TABLE 1:** Settings used for the graph layout of Figure 6. The nucleotide range 7744–9984 was used for the GS. The order in the table represents the genome order (GS: guide sequence, CS: comparative sequence).

Sequence	Genome	Contig	Color
GS	SenAgo	NC_011148	red
CS1	SenHei	NC_011083	green
CS2	SenNew	NC_011080	purple
CS3	SenAgo	NC_011149	pink
CS4	supergenome		brown

The red edges represent the guide sequence (GS) which traverses the alignment blocks $\left\{v_{0}\right.$ - $\left.v_{3},v_{6}-v_{8},v_{10},v_{13}-v_{18},v_{20},v_{21}\right\}$ thereby fulfilling the Requirements 1–3. The comparative sequences are ordered in the genome order (Requirement 4) with the following colors: green, purple, pink, and brown. The Requirements 5–7 are fulfilled for the comparative sequences, and the orthogonal edge layout supports the readability of the edges.

The green edges represent the first comparative sequence (CS1) having the highest priority for the comparative analysis. All vertices of the GS are traversed. All edges of the CS1 point against the reading direction of the GS and follow the reverse order of the vertices of the GS. In addition, the green directional glyphs (CS1) always point against those of the GS. This may indicate that the reversed strand of the DNA was sequenced and the actual order corresponds to the one of the GS. The difference to the GS are three potential insertions, since the vertices $v_{4}$ , $v_{5}$ , and $v_{9}$ and $v_{11}$ are traversed by the CS1 and not by the GS.

The purple edges represent the second comparative sequence (CS2). The edges and the directional glyphs of the (purple) are pointing in the same direction as the ones of the CS1; all against the general reading direction, which may indicate the sequencing of the reversed strand of DNA. Unlike the CS1, some vertices ( $v_{21}$ , $v_{18}$ , $v_{17}$ , $v_{15}$ , $v_{13}$ , $v_{8}$ , $v_{6}$ , $v_{3}$ , $v_{1}$ ) are not traversed by the CS2, indicating potential deletions compared to the GS. Additionally, there are potential inserts in the CS2, namely, $v_{18}$ and $v_{19}$ connected to $v_{20}$ .

The pink edges represent the third comparative sequence (CS3). As with the CS1 and the CS2, there are also potential insertions and deletions compared to the GS. In this case the edges all point in the general reading direction and the order of the vertices shared by the GS and the CS3 follows the order of the GS. The directional glyphs always point in the same direction in the shared vertices.

The brown edges represent the fourth comparative sequence (CS4) and show a different pattern than the other comparative sequences. This is the artificial common coordinate system (supergenome) (Gärtner et al., 2018). There are regions where the CS4 and the GS are linear to each other: $\left\{v_{1}-v_{3}\right\}$ or $\left\{v_{15}-v_{18},v_{20},v_{21}\right\}$ , but there are also very erratic areas where the CS4 differs strongly from the GS: e.g., $\left\{v_{6},v_{8},v_{9},v_{7},v_{10}\right\}$ or $\left\{v_{21},v_{19},v_{0},v_{12},v_{14}\right\}$ .

### 3.2 Example 2

In the second example, a longer nucleotide interval of over 106 kb was selected as the GS. The genome order chosen as well as the coloring assigned to the sequences and the selected range are presented in Table 2.

**TABLE 2:** Settings used for the graph layout of Figure 6 (B). The nucleotide range 4,515,228–4,621,763 was used for the GS (over 106 kb). The order in the table represents the genome order (GS: guide sequence, CS: comparative sequence).

Sequence	Genome	Contig	Color
GS	SenAgo	NC_011149	red
CS1	SenHei	NC_011083	green
CS2	SenNew	NC_011080	purple
CS3	SenDub	NC_011205	pink
CS4	supergenome		brown

The comparative sequences CS2, CS3, and CS4 are mostly co-linear to the GS. All three sequences visit the same vertices in the selected area in the same order and have the same directional glyphs. With the exception of four insertions ( $v_{5}$ , $v_{8}$ , $v_{11}$ , $v_{15}$ ), where $v_{11}$ and $v_{15}$ are merged vertices with 2 and 11 alignment blocks, respectively, the order of the vertices corresponds to the GS.

However, the green sequence (CS1) is similar to the GS in some parts (it traverses all of its vertices), but it differs from the GS in terms of the order of the vertices. The CS1 starts at vertex $v_{10}$ , recognizable by the green start glyph. The sequence then proceeds in the opposite vertex order compared to the other sequences (GS, CS2, CS3, and CS4) from $v_{10}$ to $v_{4}$ (with the insertions of the other CS). The green directional glyphs of CS1 are in this section also always opposite to the glyphs of the other sequences. This is followed by an insertion, which only exists in CS1, with 3667 alignment blocks. Finally, the first three vertices of the GS followed by the last four vertices of the GS are traversed colinearly to the GS.

All these findings allow the domain users to confirm, to reject, or to purpose hypotheses regarding the comparative analysis of genomes.

## 4 Discussion and summary

For layouting gMSA graphs, we presented a complete framework together with the respective algorithms for each of the steps of the framework. Our framework is based on the Sugiyama framework, and we used or adapted existing algorithms, where possible, and developed new algorithms, where needed, within this framework. The layout obtained meets the design requirements derived from the task of visually comparing genome-wide Multiple-Sequence-Alignment (gMSA) graphs. It supports analysts in gaining insights into the closeness and distance of species based on these gMSAs. In addition, artificial common coordinate reference systems, such as the supergenome of Gärtner et al. (Gärtner et al., 2018), can be visualized. As a further possible application, this visualization can be used to visually evaluate the quality of sequence assembling or such artificial reference systems. It could also be tested to visualize pangenomes with this graph layout.

One limiting factor is the runtime of the framework. With large graphs, the time complexities, which are sometimes quadratic or even cubic for the individual steps, can result in a long runtime. Also, with a strong fragmentation of the subsequences of the alignments and strong dissimilarities between the orders, the graph can be very inflated and it can be difficult to follow the orders due to many long edges. However, this is usually due to poor alignment quality. The choice of the order of the comparative sequences has a strong influence on the graph layout. Whether a phylogenetic hierarchy or a graph-based similarity metric leads to better results for such an order has not yet been tested but would be exciting to investigate.

We have implemented a prototype of the described framework. The created graph layout can be used as a starting visualization for an exhaustive analysis of gMSAs. For a more precise analysis, further visualizations such as local MSA visualizations and visualizations of associated annotation data must of course be integrated into connected views. Our next step is therefore to expand the prototype into a visualization system with such functionalities. The final drawing could also be customized according to specific tasks. For example, the aligned sequences could be displayed directly in the vertices or additional information, such as the alignment quality, could be encoded using the vertex color. The layout created can be strongly influenced by changing the drawing parameters. In the Supplementary Figure S7, you can see a clearer encoding of the alignment block length by greatly increasing the maximum vertex width.

## Data Availability

The source code for this project is available in this repository: https://github.com/jeremias-schebera/gMSA-Graph-Browser---Source-Code.git. An executable prototype is available under the following DOI:https://doi.org/10.5281/zenodo.10284921. Docker Compose is required to execute the prototype. Both the source code and the prototype are open source under the MIT license.
