# Supplementary material for: A layout framework for genome-wide multiple sequence alignment graphs
Source: Front Bioinform. 2024 Aug 16;4:1358374. doi: 10.3389/fbinf.2024.1358374 (PMC11362851; doi:10.3389/fbinf.2024.1358374)
Supplement: Supplementary file 1 [file DataSheet1.pdf]

Supplementary Material

1 DATA FLOW DIAGRAM

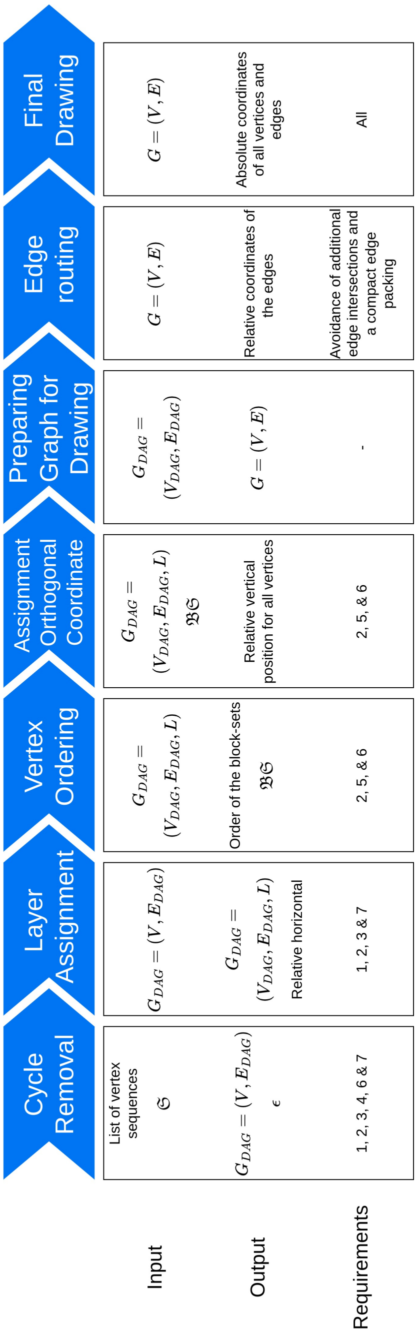

Figure S1. Data flow diagram of the framework with input, output and considered requirements.

2 CYCLE REMOVAL

2.1 Pseudo Code

The three algorithms for adding the guide sequence (Algorithm 2), a bypath in its original direction (Algorithm 3), and a bypath in its reversed direction (Algorithm 4) are rather similar. Therefore, we highlighted changes in blue and additions in green.

Algorithm 1: cycleRemoval

Input: List of vertex sequences $\mathfrak{S}$ in the order of importance (genome order) starting with the guide sequence

Result: A DAG $G_{DAG} = (V, E_{DAG})$ created from the gMSA graph and a mapping ϵ of edges of the DAG to the original edges of the gMSA graph

begin
- $initialize\ \sigma, \tau, \epsilon$;
- $GS = \mathfrak{S}[0]$;
- $addGuideSequence(GS, \sigma, \tau, V_p, E_{DAG}, \epsilon)$
- **for** $1 \leq i \leq |\mathfrak{S}| - 1$ **do**
  - $S = \mathfrak{S}[i]$;
  - $byPathList = splitInByPaths(S, V_p)$;
  - **foreach** $BP \in byPathList$ **do**
    - $v_0 = BP[0]$; $v_n = BP[|BP| - 1]$;
    - **if** $v_n \in \sigma_{v_0}$ **then**
      - $addForward(BP, S, \sigma, \tau, V_p, E_{DAG}, \epsilon)$
    - **else**
      - $addBackward(BP, S, \sigma, \tau, V_p, E_{DAG}, \epsilon)$

Algorithm 2: addGuideSequence

Input: $GS, \sigma, \tau, V_p, E_{DAG}, \epsilon$
Result: Updated $\sigma, \tau, V_p, E_{DAG}, \epsilon$
begin
  for $0 \leq i < |GS| - 1$ **do**
    $u = GS[i];\ v = GS[i+1];$
    $e = (u, v);$
    $V_p = V_p \cup \{u\} \cup \{v\};$
    $E_{DAG} = E_{DAG} \cup \{e\};$
    $\sigma, \tau = updateConnectionFunctions(e, \sigma, \tau);$
    $\epsilon_e = \epsilon_e \cup \{(forward, GS)\};$

Algorithm 3: addForward

Input: $\boldsymbol{BP}, \boldsymbol{S}, \sigma, \tau, V_p, E_{DAG}, \epsilon$
Result: Updated $\sigma, \tau, V_p, E_{DAG}, \epsilon$
begin
  for $0 \leq i < |\boldsymbol{BP}| - 1$ **do**
    $u = \boldsymbol{BP}[i];\ v = \boldsymbol{BP}[i+1];$
    $e = (u, v);$
    if $\boldsymbol{e \notin E_{DAG}}$ **then**
      $V_p = V_p \cup \{u\} \cup \{v\};$
      $E_{DAG} = E_{DAG} \cup \{e\};$
      $\sigma, \tau = updateConnectionFunctions(e, \sigma, \tau);$
    $\epsilon_e = \epsilon_e \cup \{(forward, \boldsymbol{S})\};$

Algorithm 4: addBackward

Input: $\boldsymbol{BP}, \boldsymbol{S}, \sigma, \tau, V_p, E_{DAG}, \epsilon$
Result: Updated $\sigma, \tau, V_p, E_{DAG}, \epsilon$
begin
  for $0 \leq i < |\boldsymbol{BP}| - 1$ **do**
    $u = \boldsymbol{BP}[i];\ v = \boldsymbol{BP}[i+1];$
    $\boldsymbol{e_R = (v, u)};$
    if $\boldsymbol{e_R \notin E_{DAG}}$ **then**
      $V_p = V_p \cup \{u\} \cup \{v\};$
      $E_{DAG} = E_{DAG} \cup \{\boldsymbol{e_R}\};$
      $\sigma, \tau = updateConnectionFunctions(\boldsymbol{e_R}, \sigma, \tau);$
    $\boldsymbol{\epsilon_{e_R} = \epsilon_{e_R} \cup \{(backward, S)\}};$

2.2 Example Cycle Removal

The working of our cycle removal algorithm for gMSA graphs is illustrated for the example shown in the paper. Therefore, the results for the sets V_p and E_{DAG} as well as for the mappings σ, τ, and ϵ are shown after each vertex sequence was added. The abbreviations "GS" "CS1" and "CS2" denote the guide sequence, the comparative sequence 1, and the comparative sequence 2, respectively.

2.2.1 Adding the Guide Sequence

After processing the guide sequence (GS), we obtain the following results:

$$V_p = \{v_1, v_2, v_3, v_4, v_5, v_6\}$$

$$E_{DAG} = \{(v_1, v_2), (v_2, v_3), (v_3, v_4), (v_4, v_5), (v_5, v_6)\}$$

σ :

$$\begin{array}{ll} v_1 \mapsto \{v_2, v_3, v_4, v_5, v_6\} & v_4 \mapsto \{v_5, v_6\} \\ v_2 \mapsto \{v_3, v_4, v_5, v_6\} & v_5 \mapsto \{v_6\} \\ v_3 \mapsto \{v_4, v_5, v_6\} & v_6 \mapsto \emptyset \end{array}$$

τ :

$$\begin{array}{ll} v_1 \mapsto \emptyset & v_4 \mapsto \{v_3, v_2, v_1\} \\ v_2 \mapsto \{v_1\} & v_5 \mapsto \{v_4, v_3, v_2, v_1\} \\ v_3 \mapsto \{v_2, v_1\} & v_6 \mapsto \{v_5, v_4, v_3, v_2, v_1\} \end{array}$$

ϵ :

$$\begin{array}{ll} (v_1, v_2) \mapsto \{(forward, GS)\} & (v_4, v_5) \mapsto \{(forward, GS)\} \\ (v_2, v_3) \mapsto \{(forward, GS)\} & (v_5, v_6) \mapsto \{(forward, GS)\} \\ (v_3, v_4) \mapsto \{(forward, GS)\} & \end{array}$$

2.2.2 Adding the Comparative Sequence 1

After processing the comparative sequence 1 (CS1), we obtain the following results:

$$V_p = \{v_1, v_2, v_3, v_4, v_5, v_6, \boldsymbol{v_7}, \boldsymbol{v_8}\}$$

$$E_{DAG} = \{(v_1, v_2), (v_2, v_3), (v_3, v_4), (v_4, v_5), (v_5, v_6), \boldsymbol{(v_2, v_7), (v_7, v_6), (v_1, v_8), (v_8, v_6)}\}$$

σ :

$$\begin{array}{ll} v_1 \mapsto \{v_2, v_3, v_4, v_5, v_6, \boldsymbol{v_7}, \boldsymbol{v_8}\} & v_5 \mapsto \{v_6\} \\ v_2 \mapsto \{v_3, v_4, v_5, v_6, \boldsymbol{v_7}\} & v_6 \mapsto \emptyset \\ v_3 \mapsto \{v_4, v_5, v_6\} & \boldsymbol{v_7 \mapsto \{v_6\}} \\ v_4 \mapsto \{v_5, v_6\} & \boldsymbol{v_8 \mapsto \{v_6\}} \end{array}$$

τ :

$$v_1 \mapsto \emptyset \qquad v_5 \mapsto \{v_4, v_3, v_2, v_1\}$$
$$v_2 \mapsto \{v_1\} \qquad v_6 \mapsto \{v_5, v_4, v_3, v_2, v_1, \boldsymbol{v_7}, \boldsymbol{v_8}\}$$
$$v_3 \mapsto \{v_2, v_1\} \qquad \boldsymbol{v_7 \mapsto \{v_2, v_1\}}$$
$$v_4 \mapsto \{v_3, v_2, v_1\} \qquad \boldsymbol{v_8 \mapsto \{v_1\}}$$

ϵ :

$$(v_1, v_2) \mapsto \{(forward, GS)\} \qquad \boldsymbol{(v_2, v_7) \mapsto \{(forward, CS1)\}}$$
$$(v_2, v_3) \mapsto \{(forward, GS)\} \qquad \boldsymbol{(v_7, v_6) \mapsto \{(forward, CS1)\}}$$
$$(v_3, v_4) \mapsto \{(forward, GS)\} \qquad \boldsymbol{(v_1, v_8) \mapsto \{(backward, CS1)\}}$$
$$(v_4, v_5) \mapsto \{(forward, GS)\} \qquad \boldsymbol{(v_8, v_6) \mapsto \{(backward, CS1)\}}$$
$$(v_5, v_6) \mapsto \{(forward, GS)\}$$

2.2.3 Adding the Comparative Sequence 2

After processing the comparative sequence 2 (CS2), we obtain the final results (the changes are highlighted in $\boldsymbol{green}$):

$$V_p = \{v_1, v_2, v_3, v_4, v_5, v_6, v_7, v_8, \boldsymbol{v_9}\}$$

$$E_{DAG} = \{(v_1, v_2), (v_2, v_3), (v_3, v_4), (v_4, v_5), (v_5, v_6),$$
$$(v_2, v_7), (v_7, v_6), (v_1, v_8), (v_8, v_6), \boldsymbol{(v_8, v_9), (v_9, v_7)}\}$$

σ :

$$v_1 \mapsto \{v_2, v_3, v_4, v_5, v_6, v_7, v_8, \boldsymbol{v_9}\} \qquad v_6 \mapsto \emptyset$$
$$v_2 \mapsto \{v_3, v_4, v_5, v_6, v_7\} \qquad v_7 \mapsto \{v_6\}$$
$$v_3 \mapsto \{v_4, v_5, v_6\} \qquad v_8 \mapsto \{v_6, \boldsymbol{v_9}, \boldsymbol{v_7}\}$$
$$v_4 \mapsto \{v_5, v_6\} \qquad \boldsymbol{v_9 \mapsto \{v_7, v_6\}}$$
$$v_5 \mapsto \{v_6\}$$

τ :

$$v_1 \mapsto \emptyset \qquad v_6 \mapsto \{v_5, v_4, v_3, v_2, v_1, v_7, v_8, \boldsymbol{v_9}\}$$
$$v_2 \mapsto \{v_1\} \qquad v_7 \mapsto \{v_2, v_1, \boldsymbol{v_9}, \boldsymbol{v_8}\}$$
$$v_3 \mapsto \{v_2, v_1\} \qquad v_8 \mapsto \{v_1\}$$
$$v_4 \mapsto \{v_3, v_2, v_1\} \qquad \boldsymbol{v_9 \mapsto \{v_8, v_1\}}$$
$$v_5 \mapsto \{v_4, v_3, v_2, v_1\}$$

ϵ :

$$(v_1, v_2) \mapsto \{(forward, GS), \boldsymbol{(backward, CS2)}\}$$
$$(v_2, v_3) \mapsto \{(forward, GS)\}$$
$$(v_3, v_4) \mapsto \{(forward, GS), \boldsymbol{(forward, CS2)}\}$$
$$(v_4, v_5) \mapsto \{(forward, GS), \boldsymbol{(forward, CS2)}\}$$
$$(v_5, v_6) \mapsto \{(forward, GS), \boldsymbol{(forward, CS2)}\}$$
$$(v_2, v_7) \mapsto \{(forward, CS1), \boldsymbol{(backward, CS2)}\}$$
$$(v_7, v_6) \mapsto \{(forward, CS1)\}$$
$$(v_1, v_8) \mapsto \{(backward, CS1)\}$$
$$(v_8, v_6) \mapsto \{(backward, CS1), \boldsymbol{(backward, CS2)}\}$$
$$\boldsymbol{(v_8, v_9) \mapsto \{(forward, CS2)\}}$$
$$\boldsymbol{(v_9, v_7) \mapsto \{(forward, CS2)\}}$$

2.3 Distinction from Ear Decomposition

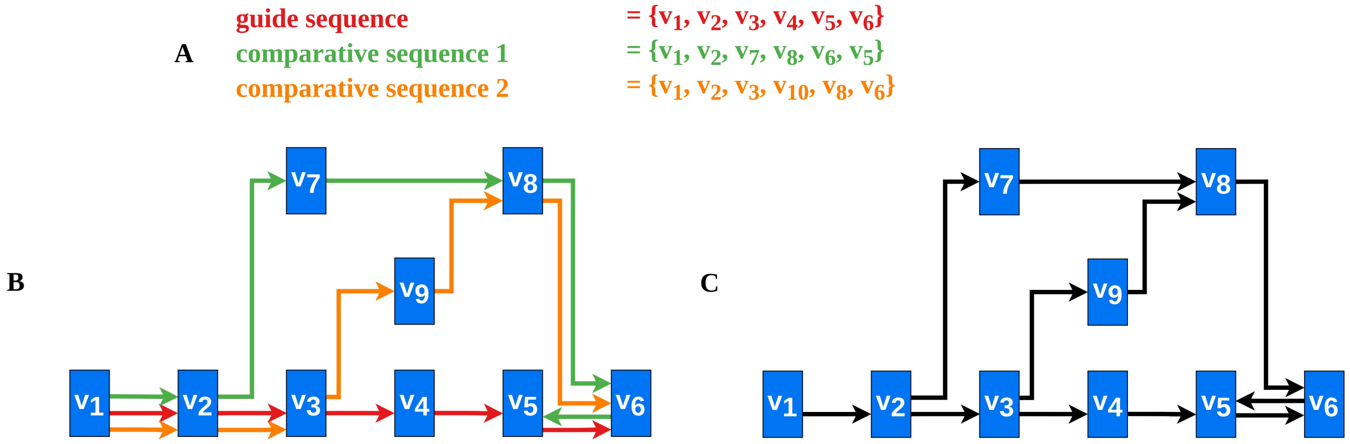

Figure S2. In this example we will show that there is not always an ear decomposition for our graphs. **(A)** The vertex sequences of the selected sequences. This is the data basis for the graph created in this example. **(B)** The graph representation of the data from (A). **(C)** The directed graph of (B) without multiple edges between the same vertices on which the ear decomposition is to be applied. The first path in the ear decomposition must be a cycle. Therefore, the first ear can only be $P_0 = \{v_5, v_6, v_5\}$, because there is only this one trivial cycle in the graph. Moreover, the graph cannot be decomposed into more ears and therefore an ear decomposition is not possible on this graph.

Although the algorithm described is a decomposition into individual paths, it is not an *ear decomposition*. The definitions we use are based on Bang-Jensen and Gutin (2002) using the term “edge” instead of the term “arc”. An *ear decomposition* is used to decompose a directed multi-graph $D = (V, E)$ with at least 2 vertices into a sequence of t *ears* $\mathcal{E} = \{P_0, P_1, P_2, ..., P_t\}$. Thereby the first ear P_0 is a cycle and each P_i is a path or a cycle with the following three characteristics:

- P_i and P_j are edge-disjoint for $i \neq j$.
- For each $i = 1, .., t$: If P_i is a cycle, then it has exactly one vertex with $V(D_{i-1})$ in common. Otherwise the start- and the end-vertex of P_i are distinct vertices of $V(D_{i-1})$ and no other vertex of P_i belongs to $V(D_{i-1})$. Note: Here, D_i is a sub-graph of D with all vertices of the ears P_0 to P_i: $V(D_i) = \bigcup_{j=0}^{i} V(P_j)$
- The edges of all ears P_0 to P_t form the set of edges of D: $E = \bigcup_{j=0}^{t} E(P_j)$

A directed multi-graph D with at least two vertices is strongly connected if and only if it has an ear decomposition. If D is strongly connected then every cycle in D can be used as start-cycle/-ear P_0 for the ear decomposition.

However, in our case the graph does not need to be strongly connected. In fact, our decomposition can also applied to graphs without any cycles at all. This is shown using an example in Figure S2.

Moreover, the aim of the two decompositions is totally different. We want to remove cycles and thereby prepare the graph for the layering step in such a way that our requirements are fulfilled, while the ear decomposition is used to check for graphs being strongly connected.

2.4 Complexity Analysis

Let $v = |V|$ denote the number of vertices and let $s = |\mathfrak{S}|$ be the number of vertex sequences in the gMSA graph. The overall time complexity of our newly developed cycle removal algorithm is $O(s \cdot v^3)$. The Tables S1 & S2 show the details of our analysis. The function $updateConnectionFunctions$ has a time complexity of $O(v^2)$ and dominates the time complexity of the Algorithms 2-4. Together with the surrounding loop, this gives the overall time complexity of $O(v^3)$ of each of these algorithms.

Line	Time complexity	Comments
2	$O(1)$	HashMap create
3	$O(1)$	ArrayList get
4	$\boldsymbol{O(v^3)}$	Algorithm 2
5	$O(s-1)$	ArrayList iterate
6	$O(1)$	ArrayList get
7	$O(v)$	splitInByPaths
8	$O(1)$	is included in Algorithm 2, Line 2; Algorithm 3, Line 2; Algorithm 4, Line 2
9	$O(1)$	ArrayList get
10	$O(1)$	HashSet contain
11	$O(v^3)$	Algorithm 3
13	$O(v^3)$	Algorithm 4
5-13	$\boldsymbol{O((s-1) \cdot v^3)}$	
Overall	$\boldsymbol{O(s \cdot v^3)}$	

Table S1. Time complexity analysis of Algorithm 1

Line		Time complexity	Comments
Algorithm 2	Algorithms 3 & 4		
2	2	$O(v)$	ArrayList iterate
3	3	$O(1)$	ArrayList get
4	4	$O(1)$	Edge create
	5	$O(1)$	HashSet contain
5	6	$O(1)$	HashSet add
6	7	$O(1)$	HashSet add
7	8	$O(v^2)$	updateConnectionFunctions
8	9	$O(1)$	HashSet add
2–8	2–9	$\boldsymbol{O(v^3)}$	
Overall		$\boldsymbol{O(v^3)}$	

Table S2. Time complexity analysis of Algorithms 2, 3, 4.

3 LAYER ASSIGNMENT

The longest-path algorithm assigns all vertices without outgoing edges (sinks) to the layer 1. If all immediate successors of a vertex have been assigned to a layer then that vertex is assigned the lowest of the layers of its immediate successors plus one (Healy and Nikolov, 2013). Thus, gradually all nodes are assigned to one level. Algorithm 5 shows a possible implementation of the longest path algorithm (Healy and Nikolov, 2013). U is the set of all vertices already assigned to a layer and Z is the set of all vertices assigned to a layer below the current layer. A new vertex v to be assigned to the current layer is picked among the vertices which have not been already assigned to a layer, i.e., $v \in V \setminus U$, and which have all their immediate successors already assigned to the layers below the current one, i.e., $N^{+}(v) \subseteq Z$ (Healy and Nikolov, 2013).

Algorithm 5: longestPath Healy and Nikolov (2013)

Input: $G_{DAG} = (V, E_{DAG})$
Result: $G_{DAG} = (V_{DAG}, E_{DAG}, L)$
begin
    $U \leftarrow \emptyset$;
    $Z \leftarrow \emptyset$;
    $currentLayer = 1$;
    while $U \neq V_{DAG}$ **do**
        select vertex $v \in V_{DAG} \setminus U$ with $N^{+}(v) \subseteq Z$;
        if *no vertex v selected* **then**
            $currentLayer = currentLayer + 1$;
            $Z \leftarrow Z \cup U$;
        else
            assign v to layer $L_{currentLayer}$;
            $U \leftarrow U \cup v$;

This algorithm has a *linear* time complexity (Mehlhorn, 1984) and is simple to implement. Further, the resulting layering has a minimum height (Healy and Nikolov, 2013).

4 VERTEX ORDERING

The following formal explanations are based on Bachmaier et al. (2010). Let $N^-(v) = \{u \in V_{DAG} | (u, v) \in E_{DAG}\}$ and $N^+(v) = \{w \in V_{DAG} | (v, w) \in E_{DAG}\}$ be the *incoming neighbors* and the *outgoing neighbors* of vertex $v \in V_{DAG}$, respectively. In an ordered properly layered graph, the sets $N^+(\cdot)$ and $N^-(\cdot)$ are sorted according to the order of the vertices in the respective layers. The layers on which the vertices of the block B are placed are retrieved by $layers(B)$. For a block B, the *rightmost vertex* $x = right(B)$ is the vertex with no incoming edges from B, while the *leftmost vertex* $x = left(B)$ is the vertex with no outgoing edges from B. In the case of a single vertex block $x = y$. The terms $right(B)$ and $left(B)$ are equivalent to the terms $upper(B)$ and $lower(B)$ introduced by Bachmaier et al. (2010) since our graph layout is rotated by 90 degrees. With $l(v)$ the layer of the vertex v is retrieved.

4.1 Algorithms

4.1.1 Global sifting

Algorithm 6: globalSifting

Input: Properly k-layered graph $G_{DAG} = (V_{DAG}, E_{DAG})$, number r of sifting rounds
Result: Ordered list of all block-sets $\mathfrak{BS}$ of G_{DAG}

```
1 begin
2     create list 𝔅𝔖 of all block-sets in G_DAG;
3     for 1 ≤ i ≤ r do
4         foreach BS ∈ 𝔅𝔖 do
5             𝔅𝔖 = siftingStep(𝔅𝔖, BS);
6     return 𝔅𝔖
```

The method for global sifting is described by Algorithm 6. During r sifting rounds, all block sets are processed successively (Lines 3–5). The current *sifting block-set* BS is processed by the function $siftingStep$ (Line 5; described in detail in Section 4.1.2, Algorithm 7). After the r sifting rounds, the sequence of the block-sets is returned that has a heuristically calculated minimum of edge crossings while considering our requirements.

4.1.2 Sifting step

Algorithm 7: siftingStep

Input: Ordered list of all block-sets $\mathfrak{BS}$ of G_{DAG}, block-set $BS \in \mathfrak{BS}$ to sift
Result: Ordered list of all block-sets $\mathfrak{BS}$ with updated odering

1 **begin**
2 $\quad \mathfrak{BS}' = BS \prec \mathfrak{BS}[0] \prec ... \prec \mathfrak{BS}[|\mathfrak{BS}| - 1]$;
3 $\quad$ sortAdjacencies($\mathfrak{BS}'$);
4 $\quad x = 0; x^* = 0;$
5 $\quad p^* = 0;$
6 $\quad$ **for** $1 \leq p \leq |\mathfrak{BS}'| - 1$ **do**
7 $\quad\quad x = x+$siftingSwap($BS, \mathfrak{BS}'[p]$);
8 $\quad\quad$ **if** $x < x^*$ **then**
9 $\quad\quad\quad x^* = x; p^* = p;$
10 $\quad$ **return** $\mathfrak{BS}'[1] \prec ... \prec \mathfrak{BS}'[p^*] \prec BS \prec \mathfrak{BS}'[p^* + 1] \prec ... \prec \mathfrak{BS}'[|\mathfrak{BS}'| - 1]$

The sifting step proposed by Bachmaier et al. (2010) (Algorithm 3) was adapted for being able to handle block-sets. During the sifting step (Algorithm 7), the optimal position—the position with the minimum number of edge crossings—of a sifting block-set BS within its block-set list $\mathfrak{BS}$ is determined. The updated block-set list $\mathfrak{BS}'$ with the sifting block-set BS placed at its optimal position is returned.

Before sifting, the variables are initialized (Lines 2–5). The sifting block-set BS is placed at the first position of the temporary block-set list $\mathfrak{BS}'$ (Line 2). Using the function $sortAdjacencies$ (Line 3; described in detail in Section 4.1.3, Algorithm 8), the vertical positions of the block-sets and the adjacency data structures of the graph are initialized. Then, the variables x (*current number of edge crossings*), x^* (*best number of edge crossings*), and p^* (*best block-set position*) are initialized (Lines 4 and 5). Since the edge crossing calculation is performed relative to the start position of the sifting block-set BS, the current number of edge crossings is always set to zero at the beginning, regardless of the absolute number of crossings.

The sifting block-set BS is consecutively swapped with all other block-sets from the block-set list $\mathfrak{BS}$ by the function $siftingSwap$ (Line 7; described in detail in Section 4.1.4, Algorithm 9). Thereby, the change in the number of crossings x is calculated and updated. Moreover, the best number of edge crossings x^* and the best block-set position p^* are updated, if a smaller number of edge crossings was achieved using the current position of BS (Lines 8 and 9).

4.1.3 Sort adjacencies

Algorithm 8: sortAdjacencies

Input: Ordered list of all block-sets $\mathfrak{BS}$ of G_{DAG}
Result: Ordered sets $N^{\cdot}(B)$ and $I^{\cdot}(B)$ for each block $B \in BS$ of each block-set $BS \in \mathfrak{BS}$

1 **begin**
2     $pos = 0$;
3   **for** $0 \leq i \leq |\mathfrak{BS}| - 1$ **do**
4   **foreach** $B \in \mathfrak{BS}[i]$ **do**
5             $\pi(B) = pos$;
6   clear arrays $N^{\cdot}(B)$ and $I^{\cdot}(B)$;
7   pos++;
8         $\eta(\mathfrak{BS}[i]) = i$;
9   **foreach** $BS \in \mathfrak{BS}$ **do**
10   **foreach** $B \in BS$ **do**
11   **foreach** $e \in \{(u, v) \in E_{DAG} | v = left(B)\}$ **do**
12   add v to the next free position j of $N^{+}(u)$;
13   **if** $\pi(B) < \pi(block(u))$ **then**
14                     $p[e] = j$;
15   **else**
16                     $I^{+}(u)[j] = p[e];\ I^{-}(v)[p[e]] = j$;
17   **foreach** $e \in \{(w, x) \in E_{DAG} | w = right(B)\}$ **do**
18   add w to the next free position j of $N^{-}(x)$;
19   **if** $\pi(B) < \pi(block(x))$ **then**
20                     $p[e] = j$;
21   **else**
22                     $I^{-}(x)[j] = p[e];\ I^{+}(w)[p[e]] = j$;

The index arrays $N^{\cdot}(B)$ and $I^{\cdot}(B)$ were introduced by Bachmaier et al. (2010) for accelerating the sifting swap (Section 4.1.4). Again, we extended this function such that it handles block-sets. The index arrays are created by the function $sortAdjacencies$ (Algorithm 8) as part of the preparation step of the function $siftingStep$ (Algorithm 7). The position of a block B depends on its position within the block-set BS and the position of the block-set BS in the block-set list $\mathfrak{BS}$. Therefore, the block-sets are treated in the order given by the block-set list $\mathfrak{BS}$ (Line 3, Algorithm 8) and the blocks are treated in the order given by the current block-set (Line 4, Algorithm 8).

First, a unique vertical position is set for every block B by setting the position of block B to the current position pos (Line 5), the adjacency data structures are cleared (Line 6), and the current position pos is increased by 1 (Line 7).

Then, the adjacency data structures of the graph are initialized (Lines 11–22) similarly to the approach of Bachmaier et al. (2010), the only difference being the additional outer loop taking block-sets (Line 9) in addition to blocks (Line 10) into account.

4.1.4 Sifting swap

Algorithm 9: siftingSwap

Input: Consecutive block-sets BS_s and BS_n
Result: Change in the number of crossings

1 **begin**
2 $\quad \Delta = 0;$
3 $\quad$ **for** $B_s \in BS_s[|BS_s| - 1] < ... < BS_s[1] < BS_s[0]$ **do**
4 $\quad\quad$ **foreach** $B_n \in BS_n$ **do**
5 $\quad\quad\quad L = \emptyset;$
6 $\quad\quad\quad$ **if** $l(right(B_s)) \in layers(B_n)$ **then**
7 $\quad\quad\quad\quad L = L \cup \{(l(right(B_s)), -)\}$
8 $\quad\quad\quad$ **if** $l(left(B_s)) \in layers(B_n)$ **then**
9 $\quad\quad\quad\quad L = L \cup \{(l(left(B_s)), +)\}$
10 $\quad\quad\quad$ **if** $l(right(B_n)) \in layers(B_s)$ **then**
11 $\quad\quad\quad\quad L = L \cup \{(l(right(B_n)), -)\}$
12 $\quad\quad\quad$ **if** $l(left(B_n)) \in layers(B_s)$ **then**
13 $\quad\quad\quad\quad L = L \cup \{(l(left(B_n)), +)\}$
14 $\quad\quad\quad$ **foreach** $(l, d) \in L$ **do**
15 $\quad\quad\quad\quad \Delta = \Delta + uswap(B_s, B_n, N^d(B_s), N^d(B_n))$;
16 $\quad\quad\quad\quad updateAdjacency\ (B_s, B_n, N^d(B_s), I^d(B_s), N^d(B_n), I^d(B_n))$;
17 $\quad\quad\quad \pi(B_s) = \pi(B_s) + 1; \pi(B_n) = \pi(B_n) - 1;$
18 $\quad$ swap positions of BS_s & BS_n in $\mathfrak{BS}$;
19 $\quad$ **return** Δ

The function $siftingSwap$ is an extension of the one proposed by Bachmaier et al. (2010) (Algorithm 4), too, again taking block-sets into account. It swaps the *sifting block-set* BS_s and the *next block-set* BS_n (Algorithm 9), calculates the change in the number of crossings Δ, and returns this change. Therefore, the blocks of the two block-sets are swapped in a specific order, which is illustrated in Figure S3. The blocks of BS_s are processed backwards (Line 3) and the blocks of BS_n are processed forward (Line 4). The block iterations are nested, so that each block of the sifting block-set is swapped with each block of the next block-set. Inside the inner loop (Lines 5–17), the change in the number of edge crossings Δ, if two blocks are swapped, is calculated. This is similar to the approach of Bachmaier et al. (2010), and therefore will not be described in more detail, here. This includes the functions $uswap$ (Line 15) and $updateAdjacencies$ (Line 16), which were also not adapted. Finally, the positions of the block-sets in the block-set list $\mathfrak{BS}$ are swapped (Line 18).

4.2 Complexity Analysis

The time complexity of the global k-level crossing reduction heuristic by Bachmaier et al. (2010) is quadratic in the number of edges in the DAG ($O(|E_{DAG}|^2)$). This depends mainly on swapping the blocks. Since in our new approach the blocks are only organized in block-sets, but in the core the algorithm is still swapping consecutively the blocks, the complexity remains the same and is $O(|E_{DAG}|^2)$.

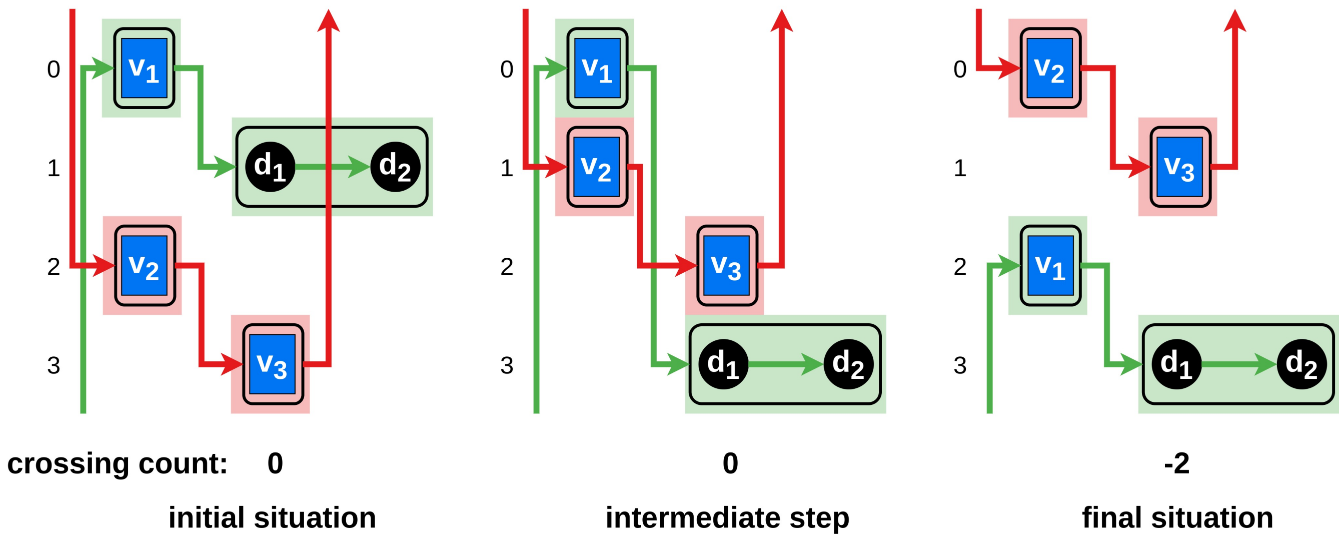

Figure S3. The blue rectangles in the graphs represent vertices and the black circles represent dummy vertices. The black frames around single vertices or sequences of dummy vertices illustrate blocks. Each block has a unique vertical position and is associated with a block-set. In this figure, there are two block-sets that are illustrated by the green and the red rectangles. The vertical swap of the green block-set with the red block-set and the corresponding crossing calculation is illustrated in two steps. In doing so, each block of the green block-set is swapped with each block of the red block-set corresponding to their order in the block-sets. The blocks in a block-set are sorted by their lowest layer. The green block-set is the sifting block-set BS_s and the block order is processed backwards. The red block-set is the next block-set BS_n and the block order is processed forward. Therefore, the last block of the green block-set is first swapped with the first block of the red block-set. The crossing number is determined relative to the initial situation, where the count is set to zero. The intermediate step shows the situation after swapping the green block-set containing the dummy vertices first with the red block-set containing v_2 and then with the red block-set containing v_3. As one intersection has been replaced by another by these swaps, the relative crossing number remains zero. After swapping the second green block-set containing v_1 first with the red block-set containing v_2 and then with the red block-set containing v_3, the crossing number is minus two, i.e., the number of edge crossings was reduced.

5 ASSIGNMENT OF THE ORTHOGONAL COORDINATE

5.1 Optimization for Special Cases

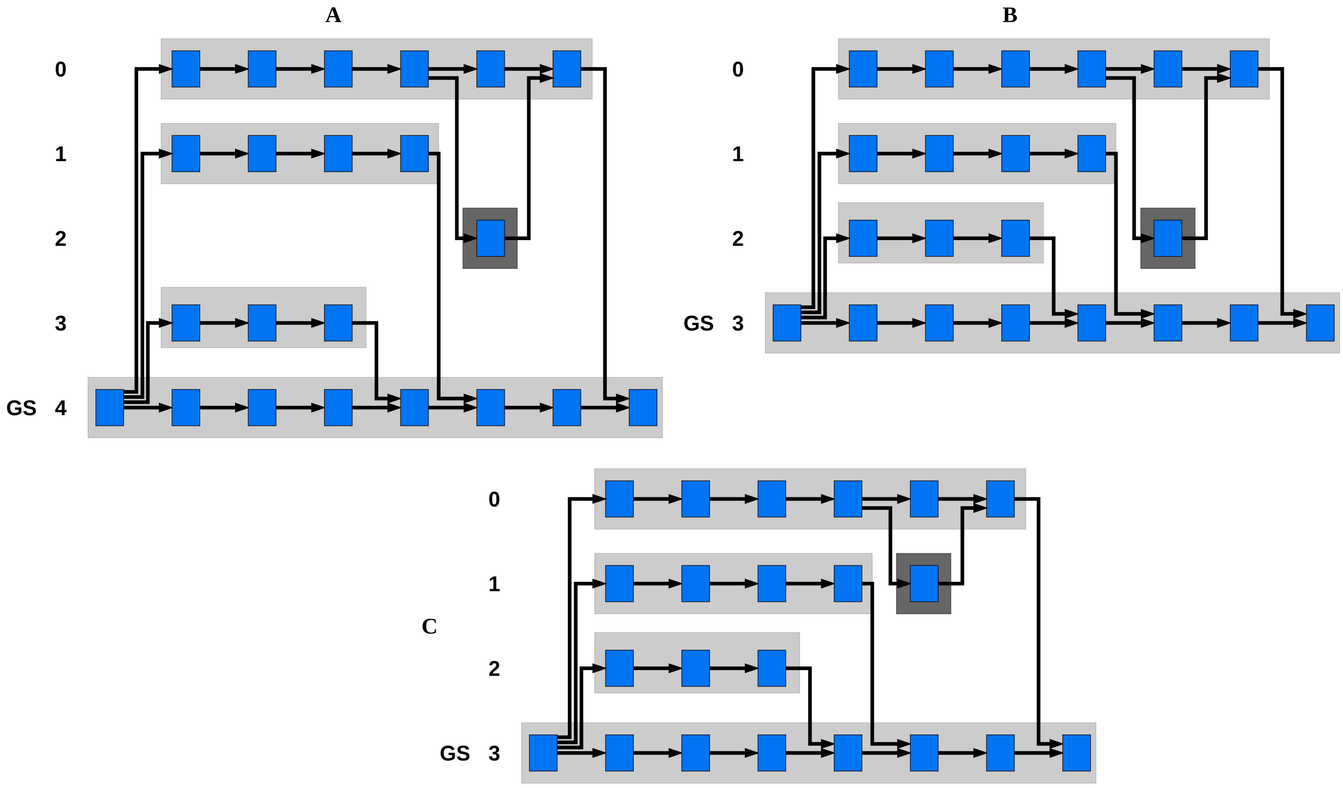

Figure S4. The blue rectangles in the graphs represent the vertices, while the gray rectangles represent block-sets. *GS* is the abbreviation for guide sequence. **(A)** Each block-set has a unique vertical position which represents the index in the block-set list $\mathfrak{B}\mathfrak{S}$ (initial situation after vertex ordering. **(B)** The vertical positions of the block-sets after the compaction. The block-sets are as close as possible to the guide sequence. The vertical position of the darker block-set is a special aesthetically unpleasing case that can be improved by the post-processing step. **(C)** The vertical position of the darker block-set was improved in a post-processing step by shifting the block-set one position upwards. Thus, this block-set is now closer to the block-sets it is connected to, vertical long edges are avoided, and the readability is improved.

After positioning the block-sets, the vertical space of the block-sets was minimized and all block-sets are as close as possible to the guide sequence. At this point, some vertical positions of the block-sets might lead to a layout that does not reflect the connection of vertices. Such a case is shown in Figure S4 (B), where the dark gray block-set is close to the guide sequence, but not connected to any of its vertices. On the other hand, it is connected to the uppermost block-set, but separated from it.

This special case can be improved in a post-processing step. It is only discussed for the block-sets above the guide sequence as the situation for block-sets below the guide sequence is symmetrical and thus can be handled accordingly. In this particular special case, the following two conditions must **both** be met, to move a block-set BS one position up:

1. The vertical positions of all block-sets connected to the block-set BS are above the vertical position of the block-set BS.

2. One vertical position above the block-set BS, none of the horizontal positions (layers) of the block-set BS may be occupied by another block-set.

The vertical positions are processed from the uppermost position to the position above the guide sequence. If there is a block-set BS at the current vertical position which meets both conditions, this block-set BS is moved up until at least one of the two conditions is no longer fulfilled. This procedure allows us to move nested special cases of this type vertically upwards one after the other. In the example of the dark highlighted block-set from Figure S4 (B) & (C), the two conditions are met. Condition 1 & 2 allows shifting the block-set at most one positions vertically upwards.

As there are countless possibilities for such improvements and detecting some of them is very complex, only one simple and important case is improved (Figure S4 (B) & (C)).

5.2 Complexity Analysis

Let $v = |V_{DAG}|$ denote the number of vertices in the DAG and let $bs = |\mathfrak{BS}|$ be the number of block-sets in the DAG. To put this in perspective, it should be noted that $bs \leq v$ but usually bs is much smaller than v. The complexity is analyzed in the order in which the algorithm is described in the paper.

To find the guide sequence in the block-set list $\mathfrak{BS}$ the complexity is $O(bs)$ since in worst case every block-set has to be checked. Every vertex of a block-set has to be processed to stack the block-set at the next free vertical position. This is done for every block-set in $\mathfrak{BS}$ and every vertex is unambiguously assigned to a block-set, therefore the complexity for this step is $O(v)$.

For the optimization of the special case, every block-set in $\mathfrak{BS}$ is processed by checking the two conditions described in Section 5.1. To check Condition 1, all connected block-sets of the current block-set have to be processed which leads to a complexity of $O(bs)$. To check Condition 2 (for one block-set) the complexity is $O(v \cdot bs)$ because in the worst case every block-set has a unique vertical position that must be checked (except the one of the guide sequence and the current block-set) by processing all vertices of this block-set. Therefore, the overall complexity for the optimization of the special case is $O(v \cdot bs^2)$.

The overall time complexity of our newly developed vertical position algorithm is also $O(v \cdot bs^2)$ since the complexity of the special case handling dominates this algorithm.

6 PREPARING THE GRAPH TO BE DRAWN

The vertices $u, v \in V_{DAG}$ are connected by a path of dummy vertices that represent a long edge $e_L \in E_L$ whereby u is the left vertex and v is the right vertex. Further $l \in V_D$ is the leftmost dummy vertex and $r \in V_D$ is the rightmost dummy vertex of the dummy path. There are 4 different possible configurations for long edges that have to be handled in different ways:

1. u and v are on the same y-position as the dummy vertices
2. u is on the same y-position as the dummy vertices, but v is on a different y-position
3. v is on the same y-position as the dummy vertices, but u is on a different y-position
4. u and v are on the same y-position, but the dummy vertices are on a different y-position

A reduction is only necessary if a dummy path contains more than one dummy vertex except for Configuration 1. In Configurations 1 only one straight edge independent of the length is necessary. Therefore, all dummy vertices and the dummy edges of such a dummy path can be replaced by one new edge (u, v) in E_{DAG}. In the Configurations 2 and 3 two edges are needed, respectively. The edges (u, r) and (r, v) replace the dummy edges in Configuration 2. Contrary, in Configuration 3 the edges (u, l) and (l, v) replace the dummy edges. In Configuration 4 the dummy edges of a dummy path are replaced by the three edges (u, l); (l, r); (r, v). The dummy vertices not used are deleted from V_{DAG}. Simultaneously, the multiple edge function ϵ is updated such that all new dummy edges are mapped to the values of the replaced dummy edge and the replaced dummy edges are removed. With this cleaning step, a lot of dummy vertices and dummy edges from G_{DAG} can be removed, which simplifies the drawing step.

6.1 Complexity Analysis

Let $v = |V_{DAG}|$ denote the number of vertices in the DAG, $v_D = |V_D|$ be the number of dummy vertices in the DAG, $e_L = |E_L|$ denote the number of long edges in the DAG, $e_D = |E_D|$ denote the number of dummy edges in the DAG, and let $e_{DAG} = |E_{DAG}|$ be the number of all edges in the DAG. To put this in perspective, it should be noted that $v_D < v$ and $e_L < e_D < e_{DAG}$.

The algorithm processes every long edge which leads to a complexity of $O(e_L)$. For every long edge, the containing dummy edges have to be removed from E_{DAG}, resulting in a complexity of $O(e_D \cdot e_{DAG})$. inserting the replacing edges in E_{DAG} and updating the function ϵ are not relevant for the time complexity. Removing the dummy vertices not used from V_{DAG}, adds a complexity of $O(v_D \cdot v_{DAG})$. This leads to a total complexity of $O(e_L \cdot (e_D \cdot e_{DAG} + v_D \cdot v_{DAG}))$. Even though this complexity has a sort of cubic influence of the edges, the resulting running time is probably more like $O(e_{DAG} + v_{DAG})$.

7 EDGE ROUTING

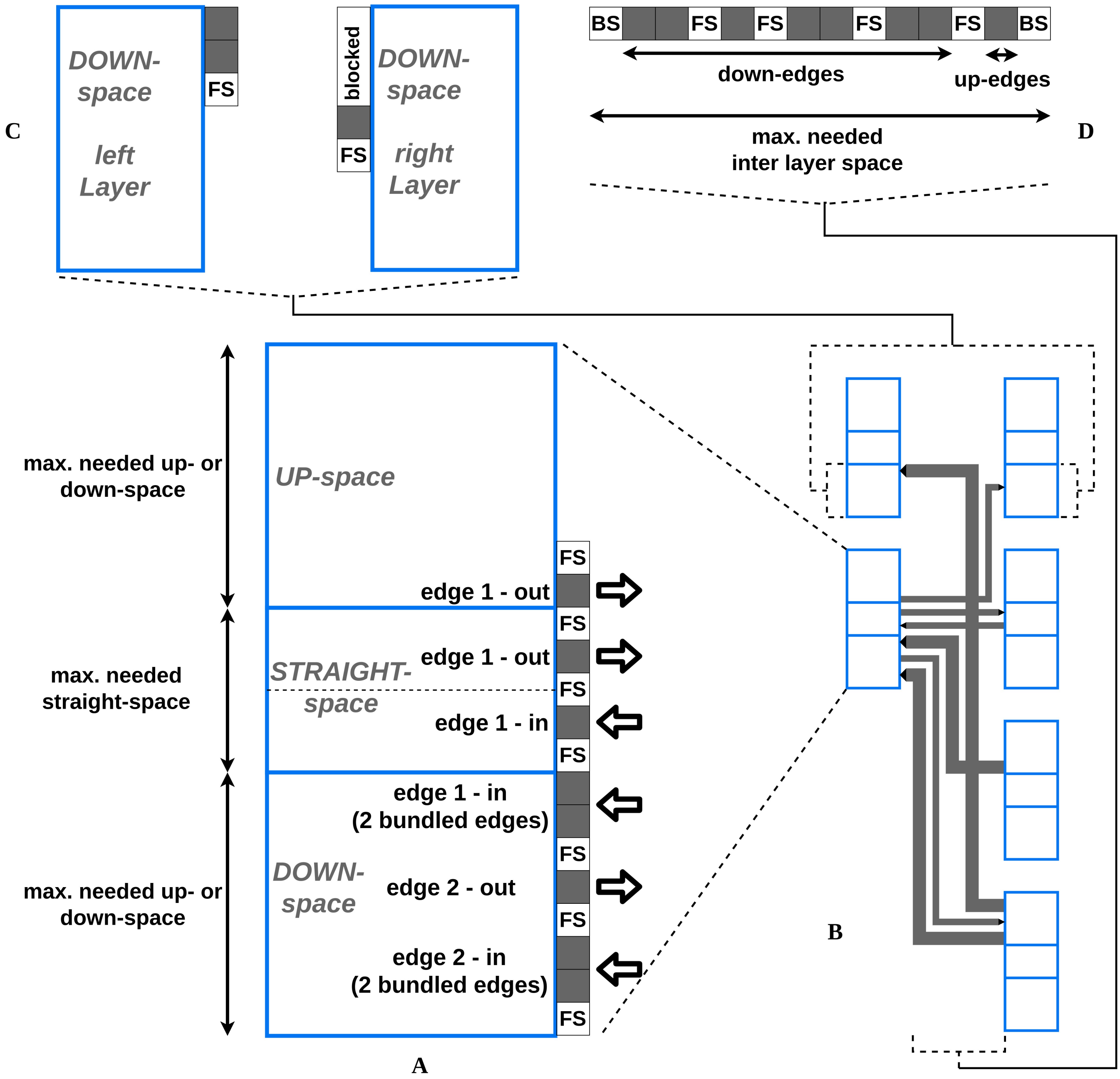

Figure S5. The blue boxes represent the vertices, whereby every vertex consists of 3 areas: the up-, the straight-, and the down-space. The dotted line in the straight-edge space of (A) illustrates the vertical center of the vertex. The gray boxes represent the edge positions. “FS” is the abbreviation for free space and represents the amount of free space between two edges. “BS” stands for border space and represents the amount of free space between the border of a vertex and the vertical line of an edge. **(A)** A detailed view of placing edges at the right border of a vertex is shown. **(B)** The final edge layout between two layers created by our approach is shown. One of the vertices is shown in (A) in detail. **(C)** The detailed view of the down-space of two opposite vertices from (B) is shown. To avoid (partial) overlaps, first the down-edges from the left vertex and afterwards the down-edges from the right vertex are placed. The space used for the down-edges of the left vertex is blocked and can not be used at the right vertex. **(D)** The detailed view of the horizontal layering of the vertical lines of all edges from (B) in the inter layer space is shown. Every vertical line of an edge has a unique horizontal position in the layout to avoid additional intersections.

7.1 Algorithm

7.1.1 Preprocessing

During the preprocessing step, first, for each layer, the vertices are sorted according to their vertical position. Then, the right and the left sides of every vertex are processed consecutively. All edges starting or ending at the right side of a vertex are categorized according to their vertical orientation to create an up-, a straight-, and a down-edge list. In the straight-edge list, there can be at most one edge in forward direction and one edge in backward direction. The edges of the up- and down-edge lists are sorted by their vertical difference. Thus, the edges in two different directions between the same vertices, which are associated to the same edge in the DAG, will be next to each other. Simultaneously in this step, the maximally needed space for the straight-edges (independent of being needed on the right or on the left border of the vertex) is calculated, to reserve this space for the straight-edges in the middle of every vertex, only (Figure S5 (A)). Therefore, the vertical space needed of the potentially existing edge in forward direction is summed with the vertical space needed of the potentially existing edge in backward direction and the corresponding free space between. The left side of a vertex is processed symmetrically.

7.1.2 Calculating relative edge-vertex connection coordinates

Calculating the relative edge-vertex connection coordinates is performed for each vertex. We describe this calculation only for the right side of a vertex, since the left side is processed symmetrically.

7.1.2.1 Vertices without Dummy Vertices

Only those vertices that are not dummy vertices ($v \in V \setminus V_D$) are processed. First, the edges from the up-edge list are placed at the up space of the vertex. Those edges are placed successively according to their vertical differences, where the edge with the smallest difference is placed closest to the straight space and the edge with the highest difference closest to the top of the vertex (Figure S5 (A)). For edges in two different directions between the same vertices (associated to the same edge in the DAG), the edge in forward direction is always placed above the edge in backward direction. A certain amount of free space “FS” separates the edges from each other. Afterwards, the straight-edges are placed in the straight-space, where the vertical center of the required straight-space is placed on the vertical center of the vertex. Finally, the edges from the down-edge list are placed at the down-space of the vertex. Like the up-edges, the down-edges are placed successively according to their vertical difference, where the edge with the smallest difference is placed closest to the straight-space and the edge with the highest difference closest to the bottom of the vertex (Figure S5 (A)). Again, the edge in forward direction is always placed above the edge in backward direction, if these edges are associated to the same edge in the DAG.

For two horizontally adjacent vertices on the same y-position having both up- or down-edges those edges in the same direction might (partially) overlap in their horizontal lines. To avoid this, first the edges from the left vertex and afterwards the edges from the right vertex are positioned at the respective vertices. The space used for the edges of the left vertex is blocked and can not be used at the right vertex (Figure S5 (C)).

Simultaneously, the height needed for every vertex (*needed vertex height*, independent whether needed on the right or the left border of the vertex) is calculated as being twice the maximally needed up- or down-space of the vertex (whichever is larger) plus the maximally needed straight-space. This information will be used during the fourth step (Section 7.1.4).

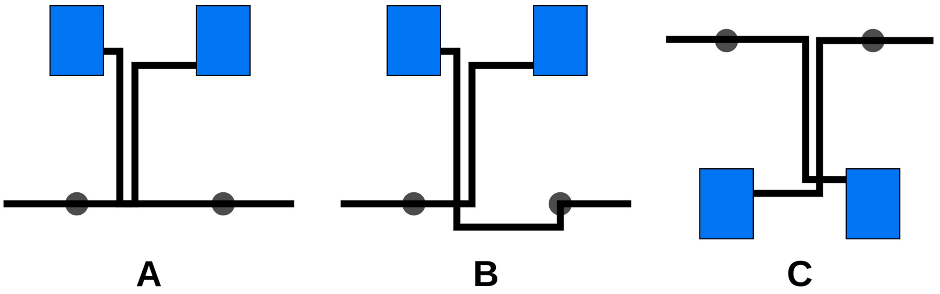

Figure S6. The blue boxes represent vertices and the gray dots represent the position of the dummy vertices. Dummy vertices are not drawn but are only important for the position of the long edges. **(A)** In the case, that two dummy vertices are on the same vertical coordinate adjacent to each other and the vertical orientation of the connected edges of both of the dummy vertices are up, the edges may overlap. **(B)** To avoid such overlapping, the connected edges of one dummy vertex are shifted a bit down. **(C)** In the case, that two dummy vertices are on the same vertical coordinate adjacent to each other and the vertical orientation of the connected edges of both of the dummy vertices are down, an overlapping of edges is not possible.

7.1.2.2 Dummy Vertices

Since the dummy vertices are not drawn in the final layout, dummy edges are handled separately for calculating the relative edge-vertex connection coordinates. This is done in order to avoid vertical differences between the left and the right edge of the dummy vertex, which would produce an offset in the long edge drawn. All connection points between the dummy vertices and the dummy edges are processed like straight edges. This leads to the edges touching each other and thus, every long edge looks like one continuous edge in the final drawing, although it consists of several parts.

There is one special case for which this procedure does not work. When two dummy vertices are on the same vertical coordinate adjacent to each other and the vertical orientation of the connected edges of both of the dummy vertices are up, the edges may overlap (Figure S6 (A)). To avoid overlapping edges in this special case, the connected edges of one dummy vertex are shifted a bit downwards (Figure S6 (B)). In other constellations with dummy vertices, overlapping edges are avoided by other mechanisms of this algorithm (e.g., Figure S6 (C)).

7.1.3 Horizontal edge layering in the inter layer space

For determining the horizontal position of the vertical lines of the edges in the inter layer space, only the edges on the right side of the left layer are considered. The two edge lists $left$ and $right$ are necessary for this step. The vertices of a layer are processed top down. For each vertex, its up-edge list E_{up} and its down-edge list E_{down} are used. The up-edge list is sorted in descending order from largest to smallest vertical difference. The down-edge list is sorted in ascending order from smallest to largest vertical difference. For every vertex v the two lists $left$ and $right$ are filled in the following way:

$$left = E_{down} \cup left \tag{S1}$$

$$right = right \cup E_{up} \tag{S2}$$

For edges in two different directions between the same vertices (associated to the same edge in the DAG), the edge in backward direction is always inserted into the list $left$ before the edge in forward direction, while the edge in forward direction is always inserted into the list $right$ before the edge in backward direction.

Afterwards, first the vertical lines of the edges of $left$ and then the vertical lines of the edges of $right$ are stacked from left to right in the inter layer space (Figure S5 (D)). A certain amount of free space "FS" separates two edges and a slightly larger amount of free space "BS" a vertex and an edge, respectively. During this step, the *needed inter layer space* is calculated. This information will be used during the fourth step (Section 7.1.4).

7.1.4 Determining the space parameter

The two space parameters, *needed vertex height* (using the information calculated during Step 2, Section 7.1.2) and *needed inter layer space* (using the information calculated during Step 3, Section 7.1.3) are calculated by searching the maximum over all vertices, respectively. These parameters are essential for obtaining a final, uniform drawing of the layout.

7.2 Complexity Analysis

Let $v = |V|$ denote the number of vertices in the gMSA graph and let $l = |L|$ be the number of layers. Since in this step the individual edges of the gMSA graph are bundled and at most only one forward and one backward edge between two vertices is processed, the number of edges in the DAG $e_{DAG} = |E_{DAG}|$ is used for the complexity analysis.

The dominant factors in the preparation step (Section 7.1.1) are:

- the sorting of the vertices per layer with a complexity of $O(v \cdot log(v))$
- and the partial sorting of the connected edges per vertex with a complexity of $O(e_{DAG} \cdot log(e_{DAG}))$, since every edge is at most processed twice in this step

Thus, the complexity of this step is $O(v \cdot log(v) + e_{DAG} \cdot log(e_{DAG}))$. The calculation of the connection coordinates between the edges and the vertices (Section 7.1.2) has a complexity of $O(e_{DAG})$, since every edge is processed twice. Determining the horizontal positions of the vertical lines of the edges in the inter layer space (Section 7.1.3) has a complexity of $O(e_{DAG})$, too. In the last step of the algorithm, the parameters are determined by processing all layers (Section 7.1.4). Therefore, the complexity for this step is $O(l)$. Since $l < v$, the complexity can be approximated overall as being $O(v \cdot log(v) + e_{DAG} \cdot log(e_{DAG}))$.

8 FINAL DRAWING - COMPLEXITY ANALYSIS

Let $v = |V|$ denote the number of vertices in the gMSA graph and let $e = |E|$ be the number of edges in the gMSA graph. The drawing of the vertices has a complexity of $O(v \cdot e)$ since the final coordinate of a vertex is added to the relative coordinates of its connected edges. The drawing of the edges has a complexity of $O(e)$.

9 DATA SET

The gMSA data set was provided by the Bioinformatic department from Leipzig University by Gärtner (2020). The data set was created with Cactus (Paten et al., 2011) for which the genomes of four Salmonella strains with their sub-structures were aligned (Table S3). Salmonella enterica serovar Agona SL483 (SenAgo) was used as the reference genome. The gMSA consists of 13,416 alignment blocks, with 50,932 sequences, combining a total number of 18,047,456 nts. With a length filter, the gMSA was cleared of 3,986 alignment blocks that were too short. Therefore, 30% of the alignment blocks are filtered out containing 0.4% of the nts in the gMSA. Thus, 9,430 alignment blocks represent this data set. The data set is stored in a Neo4j graph database.

Based on the phylogeny, the closest Salmonella strains to each other are SenNew and SenDub, followed by SenHei (Worley et al., 2018). According to Worley et al. (2018), SenAgo is in a completely different clade than the other strains and is therefore furthermost away. This should be taken into account when choosing the genome order.

In addition to the contigs in Table S3, the gMSA graph also contains an artificial order called *supergenome*, which represents an artificial global reference genome for the gMSA (Gärtner, 2020).

Name	Shortname	#Nts	Contig
S.e.s. Agona SL483	SenAgo	4836638	NC_011149 (genome)<br>NC_011148 (plasmid)
S.e.s. Dublin CT_02021853	SenDub	4917459	NC_011205 (genome)<br>NC_011204 (plasmid)
S.e.s. Heidelberg SL476	SenHei	4983515	NC_011083 (genome)<br>NC_011081 (plasmid)
S.e.s. Newport SL254	SenNew	5007719	NC_011080 (genome)<br>NC_011079 (plasmid)<br>NC_009140 (plasmid)

Table S3. Information of the Salmonella alignment. The abbreviation “S.e.s.” stands for “Salmonella enterica serovar”.

10 LAYOUT WITH STRONGER LENGTH ENCODING

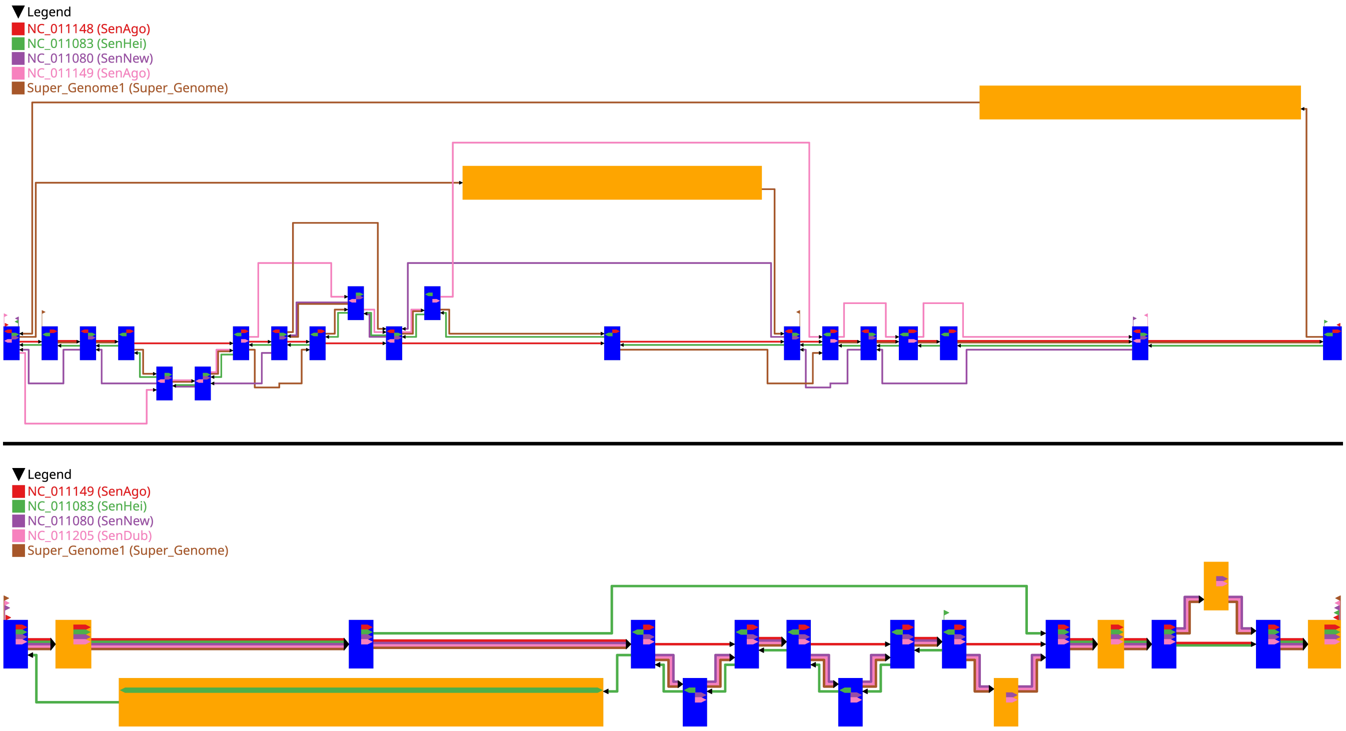

Figure S7. The layout of the graphs discussed in the 'Results' section, except that the length of the alignment blocks is coded more strongly in the vertex length. This was achieved by significantly increasing the drawing parameter for the maximum vertex widths.

REFERENCES

Bang-Jensen J, Gutin GZ. *Digraphs - theory, algorithms and applications.* (Springer) (2002), I-XXII, 1-754 .

Healy P, Nikolov SN. Hierarchical Drawing Algorithms. Tamassia R, editor, *Handbook on Graph Drawing and Visualization* (Chapman & Hall/CRC), chap. 13 (2013), 409–453.

Mehlhorn K. *Data Structures and Algorithms 2: Graph Algorithms and NP-Completeness* (Berlin, Heidelberg: Springer Berlin Heidelberg) (1984).

Bachmaier C, Brandenburg FJ, Brunner W, Hübner F. A Global k-Level Crossing Reduction Algorithm. Rahman MS, Fujita S, editors, *WALCOM: Algorithms and Computation* (Berlin, Heidelberg: Springer Berlin Heidelberg) (2010), 70–81.

Gärtner F. *Comparative Genomicsin Distant Taxa*. Dissertation, Leipzig University (2020).

Paten B, Earl D, Nguyen N, Diekhans M, Zerbino D, Haussler D. Cactus: Algorithms for genome multiple sequence alignment. *Genome research* **21** (2011) 1512–1528. doi:10.1101/gr.123356.111.

Worley J, Meng J, Allard MW, Brown EW, Timme RE. Salmonella enterica phylogeny based on whole-genome sequencing reveals two new clades and novel patterns of horizontally acquired genetic elements. *mBio* **9** (2018) e02303–18. doi:10.1128/mBio.02303-18.
